# Diversity and distribution of bamboo‐feeding true bugs in China

**DOI:** 10.1002/ece3.11563

**Published:** 2024-07-18

**Authors:** Kun Jiang, Juhong Chen, Shujing Wang, Yanfei Li, Danli Zhang, Haoyuan Hu, Wenjun Bu

**Affiliations:** ^1^ Collaborative Innovation Center of Recovery and Reconstruction of Degraded Ecosystems in Wanjiang Basin Co‐Founded by Anhui Province and Ministry of Education, School of Ecology and Environment Anhui Normal University Wuhu Anhui China; ^2^ College of Life Sciences Nankai University Tianjin China; ^3^ College of Biological Sciences and Technology Taiyuan Normal University Jinzhong China

**Keywords:** Bambusoideae, environmental factors, multiple independent origins, oligophagous insects, spatial diversity pattern

## Abstract

The Bambusoideae subfamily, originating in the late Cretaceous, has evolved to include over 1500 species globally. Notably, China hosts the richest diversity of Bambusoideae, with 728 species documented. After a long period of coevolution, plenty of animals could feed on these plants rich in cellulose and lignin. As an important group of pests and participants in the ecosystem, bamboo‐feeding true bugs (BFTBs, or bamboo‐feeding Heteropteran insects) have attracted the attention of researchers. However, the diversity and distribution of BFTBs still lack systematic and generalized research. In this study, we reviewed the BFTBs in China and simulated the diversity pattern and the driving forces of this pattern. A list of 36 genera with 69 species of BFTBs in China was obtained through paper review and field surveys. And their bamboo‐feeding habit had multiple independent origins. The spatial diversity pattern showed that the biodiversity hotspots of BFTBs are located in and around the tropics of southern China. Environmental driving force analysis showed that the minimum temperature of coldest month and annual precipitation were the dominant environmental factors shaping the spatial diversity of BFTBs. Our work quantified the diversity and distribution of BFTBs in China, providing fundamental data support for pest control and evolutionary research.

## Graphical abstract

There are 36 genera with 69 species bamboo‐feeding true bugs in China. Their spacial diversity pattern showed preference in South China, with higher diversity areas located in the tropics and its surrounding areas.

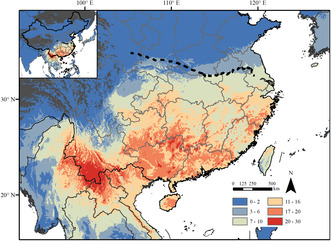

## 1 INTRODUCTION

Bambusoideae, originating from the late Cretaceous (66.89 Mya), has been documented with over 1500 species globally (Clark et al., 2015; Huang et al., 2022). These plants are naturally distributed across three regions: the African Bamboo Region, the American Bamboo Region, and the Asia‐Pacific Bamboo Region, with the latter being the most diverse, containing more than 1000 species across 58 genera. China, recognized as the most diverse country of Bambusoideae globally, harbors 43 genera and 728 species (including introduced genera and species but varieties and forms) accounting for about half of the world's genera and species of Bambusoideae (except introduced genera and species) (Shi et al., 2020; Smith & Brown, 2018; Yi et al., 2008). In addition, Chinese economic bamboo plantation covers 6.01 million hectares, accounting for 1/3 of the world's area. About 8 million tons of bamboo timber and more than 1 million tons of bamboo shoots are produced in China each year, accounting for 1/3 and 1/2 of the world's production, respectively (Qi et al., 2022; Shi et al., 2020). The extensive diversity of species and the widespread cultivation and utilization of bamboo make Bambusoideae a plant group of considerable interest in China (Chen et al., 2021; Liu, Hui, et al., 2018).

Pest management is an indispensable part of bamboo development and utilization research. Take *Hippotiscus dorsalis* as an example of Heteroptearan pest. In 1987, it caused about 800,000 moso bamboo to die in Zhejiang province and about 76% of moso bamboo died in Anji Country (Xu & Wang, 2004; Zhao et al., 2023). The calling of scientific management of bamboo pests generated many bamboo pest diversity researches in China (Huang et al., 2021; Xu & Wang, 2004; Zheng & Zou, 1982). However, Some studies may contain erroneous records of BFTBs. For example, small cabbage bug (*Eurydema dominulus*) and bean bug (*Riptortus pedestris*) are recorded to be bamboo pests (Fang & Wang, 2000; He et al., 2009). However, these two species mainly feed on Brassicaceae plants and Fabaceae plants respectively (Mizutani et al., 2011). Zheng (1994) recorded 44 bamboo‐feeding true bugs (BFTBs) according to deep field works (Zheng, 1994). It is a quite credible species list that has repeatedly been verified on field works. In addition, some pest records and new species recorded the host plants as bamboo which also credible records for BFTBs (Chen, 1989; Fan & liu, 2009). Both misleading and credible records call for updating of the BFTBs' list to apply efficient pest management policies. Furthermore, the evolutionary position and richness of different taxa of BFTBs are never well quantified. However, this evolution and richness research for these species will help to understand the origin and the different adaption ability of different taxa (family/subfamily) (Ye et al., 2022).

The Asia‐Pacific Bamboo Region has undergone the most comprehensive surveys for BFTBs distribution. *Distachys unicolor* reported by Kerzhner (1988) from South Kurile Island, north of Hokkaido, Japan (about 45° N), is the northernmost known record of BFTBs (Kerzhner, 1988). The most southeastern record is that of *Priocnemicoris cyclops* on Rossel Island, New Guinea (Brailovsky & Barrera, 2007). The westernmost record is of the *Notobitus meleagris* in Mumbai, India (72.8° E) (GBIF, National Chemical Laboratory, India). BFTBs are widely distributed within this range, roughly matching the Asia‐Pacific Bamboo Region (Yi et al., 2008). In China, Only a few bamboo species (e.g., *Phyllostachys nigra var. henonis* (Mitford) Rendle, *P. nigra* (Lodd.) Munro, *Bambusa multiplex* (Lour.) Raeusch) can distribute up to northern China (40° N) (Liu, Qiu, et al., 2018; Zheng, 1994). However, no true bugs were recorded on these bamboo. The spatial diversity of BFTBs varies within tropical and subtropical regions. For example, the distribution of *Notobitus montanus* extends north to the Qinling Mountains, but it does not reach south to Xishuangbanna, Guangdong, and southern Guangxi. However, it is in these southern regions where most *Notobitus* species are found (Jiang, Chen, & Bu, 2022; Zheng & Zou, 1982). Quantitative descriptions for BFTBs' distribution by species distribution models (SDMs) can bridge these knowledge gaps. Furthermore, the spatial diversity pattern of BFTBs and its environmental driving forces can help us to understand the formation of the diversity pattern of these oligophagous bamboo insects.

In this paper, we raised four questions: (a) How many BFTBs distributed in China? (b) How did the bamboo‐feeding habit evolve? (c) What is the spatial diversity pattern of BFTBs? (d) What kind of biodiversity hypothesis could explain this pattern? Based on our questions, we first reviewed the BFTBs recorded in published papers and books, and added new records by over 6 years of filed works in China. Then, BFTBs were marked on an evolutionary tree to explore the evolutionary history of the bamboo‐feeding habit. Finally, we used SDMs and a quite conservative method (annual temperature (AT) constraint method and point‐to‐grid method) to specify the distributions of BFTBs. Species distribution areas were then stacked to get the BFTBs spatial diversity pattern. Random forest model and generalized linear model were used to test the dominant environmental factors of this diversity pattern. Our study furnishes foundational knowledge on the diversity and distribution of BFTBs in China, facilitating pest management and evolutionary research advancement in future applications.

## 2 MATERIALS AND METHODS

### 2.1 Bamboo‐feeding true bugs diversity

Drawing on Zheng's extensively verified Chinese BFTBs list (Zheng, 1994), we synthesized this foundational data with records from additional published sources and corroborated them through our field observations. Subsequently, this reviewed list was then checked to correct the species taxonomy. From 2017 to 2023, our team conducted field collections in most provinces of China for more than 300 days and more than 5600 specimens were collected. Species were identified based on morphological characters. Combining the reviewed list and the novel records in our field works, we established the BFTBs list of China. It should be noted that our investigation is centered on true bugs predominantly feeding on bamboo. Some polyphagous species, such as *Nezara viridula*, only feeding on bamboo in rare cases were excluded. In addition, we also summarized global BFTBs by reviewing published works to augment our study. To elucidate the evolutionary position and richness pattern of BFTBs, we marked them and their richness to an evolutionary tree (Ye et al., 2022).

### 2.2 Bamboo‐feeding true bugs spatial diversity pattern

#### 2.2.1 Species occurrences

Species occurrence data were primarily sourced from specimen collections at the Institute of Entomology, Nankai University, Tianjin, China. Additional data were obtained from online databases, including the Global Biodiversity Information Facility (GBIF; https://www.gbif.org/) and iNaturalist (https://www.inaturalist.org/) and doctoral dissertations (Fan, 2011; Gao, 2010; Jiang, 2023; Yi, 2016). For records from iNaturalist, species validation was performed by examining associated images. Where only administrative locations were provided, precise coordinates were derived using Google Maps (https://www.google.com/maps). Location recorded indistinctly, out of terrestrial or limited to collection provinces or cities were excluded. To avoid spatial autocorrelation and enhance the quality of the analysis, occurrence points were spatially thinned using a 20 km buffer, employing the spThin package in R (Aiello‐Lammens et al., 2015).

#### 2.2.2 Environmental factors

Totally 19 bioclimatic factors with 2.5 arc‐min resolution from 1970 to 2000 on WorldClim 2.1 (https://worldclim.org/) were accessed as a basic environment dataset. For each species, we defined the environmental range using the minimum convex polygon method, setting a threshold of 400 km (Zhu et al., 2020). To minimize collinearity among the variables, an initial exploratory analysis of these 19 variables was conducted using default parameters. Subsequently, from pairs of variables exhibiting a high Pearson's correlation coefficient (|*p*| ≥ 0.8), only the variable with the higher area under the curve (AUC) value from a Jackknife test in the exploratory analysis was retained (Negrete et al., 2020).

#### 2.2.3 Species distribution models

Species were categorized into three groups based on the number of occurrence records (Jiang, Dong, et al., 2022). For species with five or more occurrences (*n* ≥ 5), we employed MaxEnt 3.4.4 to model their distribution areas; given that temperature has been identified as a critical environmental factor influencing insect distributions (Ahmed et al., 2016; Bale et al., 2002), we adapted our approach for species with 2 to 4 occurrences (2 ≤ *n* ≤ 4). Initially, we applied the minimum convex polygon (MCP) method with a 20 km radius to define preliminary distribution boundaries. Subsequently, we used the AT where the species occurrences covered to further constrain the species distribution area. This method was called the MCP‐AT method in this study; for the species with only 1 occurrence (*n* = 1), the pixel (5 km × 5 km) on the maps where the occurrence was located was considered as the species distribution area.

The *ENMeval* package was employed to optimize the feature class (feature class = L, H, LQ, LQH, LQHP, LQHPT) and regularization multiplier (regularization multiplier = 0.5, 1, 1.5, 2, 2.5, 3, 3.5, and 4) according to the delta AIC (Akaike information criterion). Models with minimum delta AIC, indicative of reduced parameter count and lower complexity, were considered to have superior performance (Kass et al., 2021; Muscarella et al., 2014). These optimized models were executed to predict species distribution probabilities in a logistic output format. We allocated 80% of occurrence data for training and the remaining 20% for testing, with each model configuration replicated 10 times. The bootstrap method was used to select the test set for each replication, and default settings were applied for all other parameters. Models achieving an AUC of less than 0.8 were deemed unsuccessful (Jiang, Dong, et al., 2022). Their distributions were subsequently modeled using the previously mentioned MCP‐AT method. The output was classified into three categories of habitat suitability: highly suitable (threshold: 10th percentile of training presence), moderately suitable (threshold: minimum training presence), and unsuitable.

#### 2.2.4 Spatial diversity pattern

Spatial diversity pattern was obtained by stacking all species distribution areas. For the species simulated distribution areas by MaxEnt, 10 percentile training presence was set as the threshold to get the binary distribution maps. This threshold could reduce the misestimation of the distribution areas due to imprecise location occurrence.

Species diversity distribution is not random but has a deep relationship with the environment. We evaluated 13 candidate factors based on three hypotheses: water‐energy dynamics, habitat heterogeneity, and historical climate stability (Araújo et al., 2008; Carnaval & Moritz, 2008; Hawkins et al., 2003; Simpson, 1949; Tews et al., 2004), supplemented by two variables reflecting human interference (Andermann et al., 2020; Jahani & Saffariha, 2021). These factors were categorized into five groups (Table S1). Temperature‐related, precipitation‐related, and elevation data were sourced from WorldClim, while surface roughness was obtained from EarthEnv (http://www.earthenv.org/), and the elevation range was calculated via ArcGIS which calculation method could be found in Jiang, Dong, et al., (2022). Historical climate variables (TANO and PANO) representing long‐term temperature and precipitation changes since the Last Glacial Maximum were included. Human‐related variables, population density, and built‐up surface area were sourced from the Global Human Settlement Layer (https://ghsl.jrc.ec.europa.eu/).

Random forest models and generalized linear models were applied to test the dominant environmental factors of BFTBs' spatial diversity pattern. These two models were proven to have a good ability to solve relationships between spatial diversity and environments (Jiang, Dong, et al., 2022; Li et al., 2021). Two analysis methods were used. The first method: we defined the top 5% of Chinese land areas with the highest species diversity as biodiversity hotspots (Ceballos & Ehrlich, 2006; Jiang, Dong, et al., 2022). The random forest model is not sensitive to spatial autocorrelation and multicollinearity of variables. Therefore, a random forest classification model with all 13 variables was applied to distinguish the hotspot and non‐hotspot areas. 80% data was used to train the model, with the rest 20% to test. Random forest parameters (mtry = 7 and ntree = 1000) were optimized by out‐of‐bag error (OOB error). Model performance was assessed by OOB error and AUC, MDG (Mean decrease Gini) was used to assess the relative importance of variables. The second method: the random forest regression model was applied to recognize diversity gradients by all 13 variables. The parameter of the model (mtry = 8 and ntree = 1000) was optimized by determination coefficients *R* ^2^. INP (Increase in node purity) was used to measure the relative importance of variables. We also applied generalized linear models to recognize diversity gradients by every single variable. Adjusted *R* ^2^ was used to measure the relative importance of variables (Jiang, Dong, et al., 2022; Li et al., 2021).

## 3 RESULTS

### 3.1 Species list of bamboo‐feeding true bugs

The literature review initially documented 30 genera with 53 species of BFTBs, Subsequent fieldwork added 12 genera and 18 species (Table S2). Following these efforts, our comprehensive dataset now encompasses 36 genera and 69 species of BFTBs in China (Table 1) and globally, 54 genera with 118 species (Table S3).

**TABLE 1:** Bamboo‐feeding true bugs in China.

Species	Host plants
Miridae (3 genera 4 species)	
*Mecistoscelis scirteloides* Reuter, 1891	No record
*Mystilus priamus* Distant, 1904	No record
*Elthemidea picea* Zheng, 1992	*Phyllostachys edulis* (Carriere) J. Houzeau
*E. sichuanese* Zheng, 1992	*Phyllostachys edulis* (Carriere) J. Houzeau
Blissdae (5 genera 8 species)	
*Pirkimerus japonicus* (Hidaka,1961)	*Phyllostachys edulis* (Carriere) J. Houzeau
*Macropes robustus* Zheng & Zou, 1982	No record
*M. harringtonae* Slater, Ashlock & Wilcox, 1969	*Phyllostachys edulis* (Carriere) J. Houzeau
*M. maai* Slater & Wilcox, 1973	*Phyllostachys viridis* (Young) McClure
*Iphicrates spinicaput* Scott, 1874	*Indocalamus migoi* (Nakai) P. C. Keng
*I*. *weni* Zheng, 1986	*Sinocalamus beecheyanus* (Munro) McClure
*Dimorphopterus japonicus* (Hidaka, 1959)	*Phyllostachys viridis* (Young) McClure
*Bochrus foveatus* Distant,1879	No record
Heterogastridae (1 genus 1 species)	
*Artemidorus pressus* Distant, 1903	No record
Malcidae (1 genus 1 species)	
*Malcus setosus* Stys, 1967	No record
Berytidae (1 genus 1 species)	
*Yemmalysus parallelus* Štusák, 1972	*Ampelocalamus actinotrichus* (Merr. & Chun) S.L.Chen T.H.Wen & G.Y.Sheng
Colobathristidae (1 genus 2 species)	
*Phenacantha viridipennis* Horvath, 1904	*Dinochloa utilis* McClure
*P. bicolor* (Distant,1901)	No record
Alydidae (6 genera 12 species**)**	
*Acestra sinica* Dallas 1852	No record
*A. malayana* Distant, 1903	No record
*Anacestra hirticornis* Hsiao, 1964	*Fargesia* sp.
*A. spiniger* Hsiao, 1965	No record
*Anacestra* sp.	No record
*Distachys vulgaris* Hsiao, 1964	No record
*Marcius longirostris* Hsiao, 1964	No record
*M. nigrospinosus* Ren, 1993	No record
*M. sichuananus* Ren, 1993	No record
*Marcius* sp.	No record
*Tuberculiformia subinermis* Ahmad, 1967	No record
*Paramarcius puncticeps* Hsiao, 1964	No record
Coreidae (6 genera 20 species)	
*Notobitus excellens* Distant, 1879	*Dendrocalamus* sp.
*N. sexguttatus* (Westwood, 1842)	*Bambusa textilis* McClure & *Bambusa chungii* McClure
*N. elongatus* Hsiao, 1977	No record
*N. meleagris* (Fabricius, 1787)	*Bambusa textilis* McClure & *Phyllostachys viridis* (Young) McClure
*N. montanus* Hsiao, 1963	*Phyllostachys edulis* (Carriere) J. Houzeau
*Notobitiella elegans* Hsiao, 1963	*Dendrocalamus* sp.
*N. bispina* Jiang, Chen et Bu, 2022	No record
*Cloresmus yunnanensis* Hsiao, 1963	*Dendrocalamus* sp.
*C. pulchellus* Hsiao, 1963	*Dendrocalamus* sp.
*C. modestus* Distant, 1901	*Dendrocalamus* sp.
*Cloresmus* sp1.	No record
*Cloresmus* sp2.	*Phyllostachys edulis* (Carriere) J. Houzeau
*C. similis* (Dallas, 1852)	No record
*Manocoreus marginatus* Hsiao, 1964	No record
*M. yunnanensis* Hsiao, 1964	No record
*M. vulgaris* Hsiao, 1964	No record
*M. montanus* Hsiao, 1964	No record
*M. astinus* Ren, 1983	No record
*Fracastorius cornutus* Distant, 1902	No record
*Homoeocerus striicornis* Scott, 1874	*Phyllostachys* spp.
Pentatomidae (11 genera 20 species)	
*Cressona valida* Dallas, 1851	No record
*C. divaricata* Zheng & Zou, 1982	No record
*Vitruvius insignis* Distant, 1901	No record
*Aenaria pinchii* Yang, 1934	*Phyllostachys edulis* (Carriere) J. Houzeau
*A. lewisi* (Scott,1874)	*Pleioblastus amarus* (Keng) P. C. Keng
*A. bivitta* Fan & Liu, 2009	No record
*A. zhangi* Chen, 1989	No record
*Halyabbas unicolor* Distant, 1900	*Phyllostachys edulis* (Carriere) J. Houzeau
*Brachymna tenuis* Stål, 1861	No record
*B. bificeps* Chen, 2000	No record
*B. humerata* Chen, 1989	No record
*Zouicoris elegans* Zheng,1986	No record
*Critheus lineatifrons* Stål, 1870	*Bambusa stenostachys* Hack
*C. indicus* (Distant, 1900)	No record
*Hippotiscus dorsalis* (Stål, 1869)	*Phyllostachys edulis* (Carriere) J. Houzeau
*Paterculus parvus* Hsiao & Cheng, 1977	No record
*P.elatus* (Yang, 1934)	*Pleioblastus amarus* (Keng) P. C. Keng
*P. aberrans* Distant, 1921	No record
*Dabessus albovittatus* Hsiao & Cheng, 1977	No record
*Dunnius minor* Zheng & Liu, 1987	No record

Evolutionary position and richness marking showed that the bamboo‐feeding habit of these species had multiple independent origins (Figure S1). Taxonomically, BFTBs are distributed across nine families or subfamilies, which are not closely related in a sister group context. Our richness analysis indicated that BFTBs are represented within four superfamilies: Lygaeoidea, Coreoidea, Pentatomoidea, and Miroidea, each showing varying levels of species richness. Coreoidea emerged as the most diverse, comprising 32 species distributed among three tribes: Cloresmini (13 species), Manocoreini (5 species), and Micrelytrini (12 species). The second‐most diverse is Pentatomoidea, with all 20 species belonging to the family Pentatomidae. Lygaeoidea includes 13 BFTBs across five families, with the family Blissidae representing the majority, accounting for 61.5% (8/13) of its species. Miroidea, the least diverse, contains only four species, all of which are from the family Miridae.

### 3.2 Spatial diversity pattern of bamboo‐feeding true bugs

**FIGURE 1:**
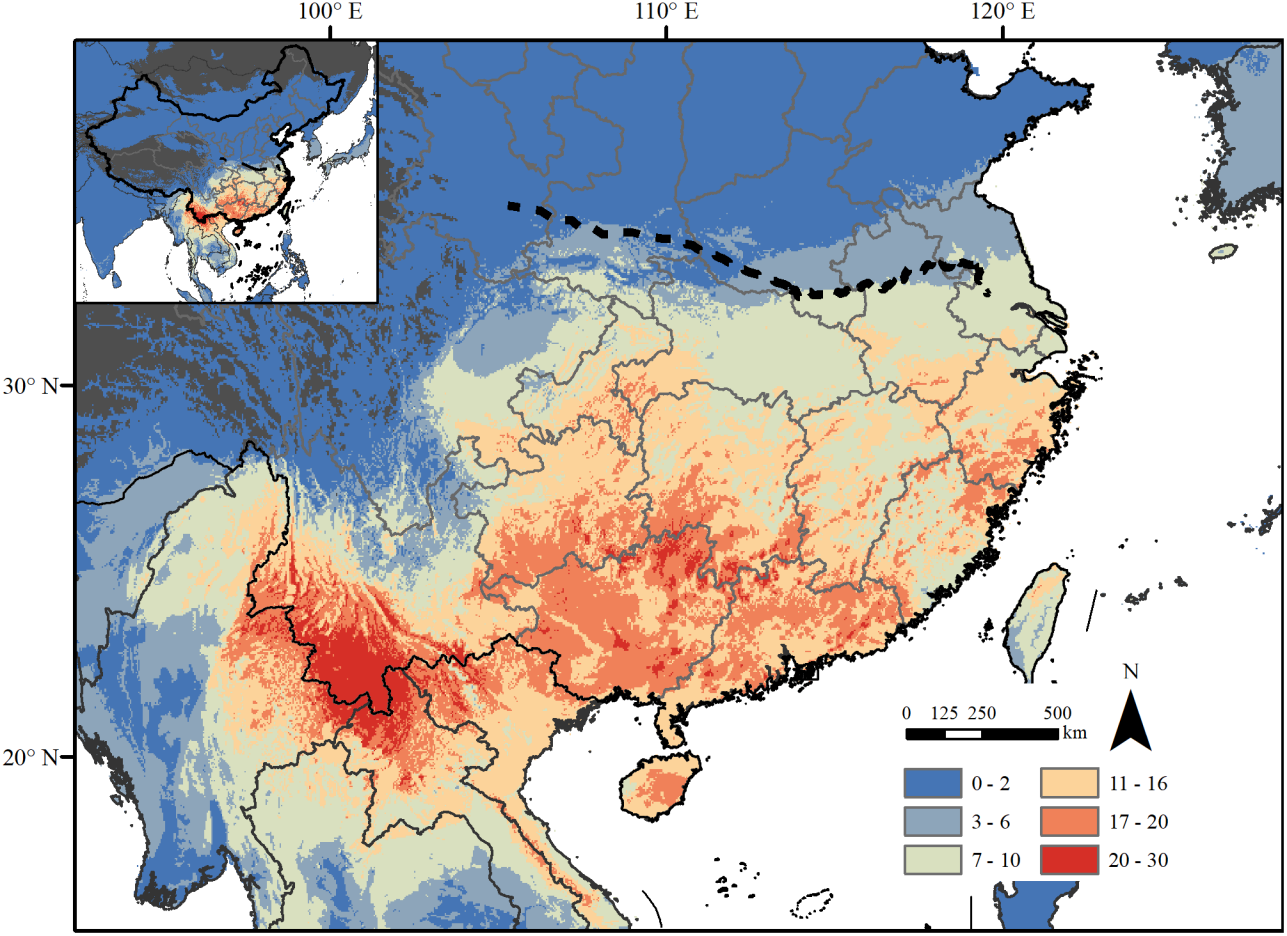
Bamboo‐feeding true bugs diversity pattern. Dark gray areas: annual mean temperature lower than 0°C; dot green line: Qingling‐Huaihe line.

MaxEnt successfully simulated the distribution areas for 30 species, the MCP‐AT method for 28 species, and the point‐to‐grid method for 11 species. Detailed results of species distribution areas and parameter selections for BFTBs with a sample size of five or more are available in the Supplementary information (Figures S2–S66). Overall, Spatial analysis indicates a significant concentration of BFTBs in Southern China, predominantly in Guangdong, Yunnan, Guangxi, Guizhou, Hainan, Hunan, Jiangxi, Fujian, Zhejiang, and the southern regions of Anhui, Jiangsu, Hubei, and Chongqing (Figure 1). Qinling‐Huaihe line and annual mean temperature 0°C isotherm line (which is also 800 mm isohyetal line), approximate the ecological boundary for BFTBs distribution. Suitable habitats for BFTBs are scarce in northern and western China. Regions such as southern Yunnan, the Pearl River Basin, and the southeast coastal area exhibit notably higher biodiversity. As biodiversity increases, areas concentrate to the tropical climate zone and its surrounding areas.

The random forest classification model showed that the average out‐of‐bag rate was 2.11%, average AUC was 0.98. The random forest regression model showed that the *R* ^2^ was 0.99 which indicated a pretty well modeling. The general linear model demonstrated the adjusted *R* ^2^ for different variables were between 0 to 0.46, which indicated that the model was not sufficiently accurate in predicting the number of species compared to the random forest model.

**FIGURE 2:**
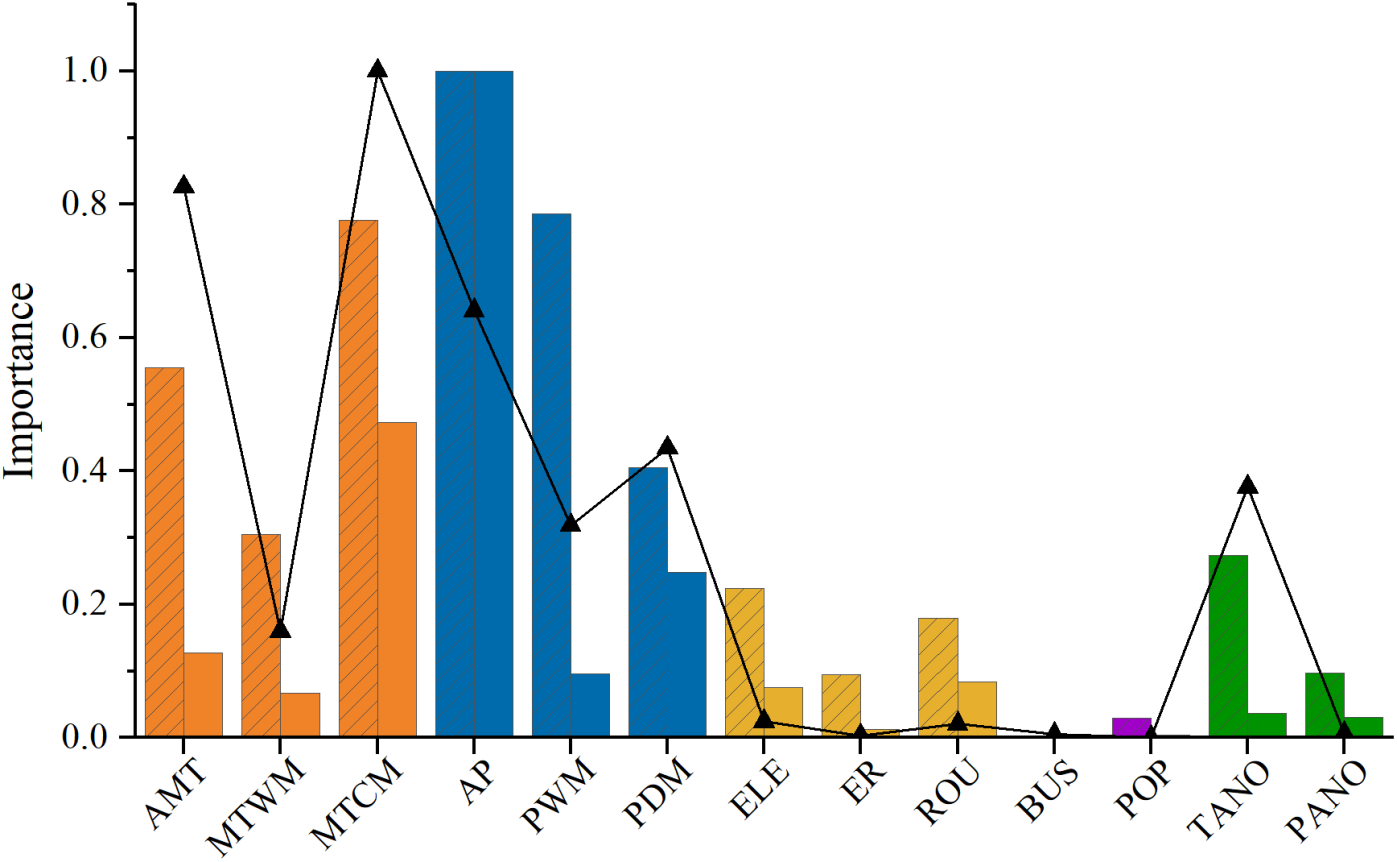
The relative importance of environmental factors for the spatial diversity pattern of bamboo‐feeding true bugs. The point and solid line diagram show the adjusted *R* ^2^ in the GLMs; The histograms with and without slashes indicate the Mean Decrease Gini (MDG) Geographically, Cloresmini is confined to the Oriental Rand Increase in Node Purity (INP) for the random forest classification and regression models, respectively. See Table S3 for definitions of abbreviations.

Given that temperature and precipitation‐related variables have been employed in modeling the distribution area of BFTBs, these variables should be divided apart from others. Among the former variables, annual precipitation was the most important variable among three different analyses, and minimum temperature of coldest month was the second most important variable which showed relatively lower values in random forest modeling. For the rest variables, TANO is more important than others. PANO combined with habitat diversity and human interference‐related variables had less impact on the spatial diversity of BFTBs (Figure 2).

## 4 DISCUSSION

### 4.1 Bamboo‐feeding true bugs diversity in China

In China, the Heteroptera encompasses approximately 2000 phytophagous species, of which those recorded in our study, termed BFTBs, represent about 3.5%. Notably, 95% of the phytophagous Heteroptera are distributed across four major superfamilies: Miridae, Pentatomoidea, Lygaeoidea, and Coreoidea (Ye et al., 2022). All BFTBs in the world fall within these superfamilies. Initially, we hypothesized that species across these superfamilies would exhibit similar propensities to feed on bamboo. Contrary to this assumption, our findings reveal pronounced disparities in the species richness among the different families/subfamilies of BFTBs.

In the Miroidea superfamily, only 1 family comprising 3 genera and 4 species has been identified as BFTBs. The Miridae family, which is the most diverse group in the Heteroptera with more than 11,300 species described, primarily comprises phytophagous species, though it includes a few predatory and zoophytophagous species as well. (Schuh & Weirauchl, 2020; Wheeler, 2001). BFTBs in this family are disproportionate to the high diversity of Miridae. This may be related to the misalignment of spatial diversity patterns between Miridae and Bambusoideae. Specifically, the diversity hotspots for Chinese endemic Miridea are primarily located in the peripheral areas of the Sichuan Basin (Jiang, Dong, et al., 2022), whereas Bambusoideae exhibit higher diversity in hydrothermally rich regions such as Guangdong, Guangxi, and Hainan (Bystriakova et al., 2003). This geographical and ecological mismatch likely contributes to the underrepresentation of BFTBs within Miridea.

In the Pentatomoidea superfamily, 1 family, 11 genera with 20 species were recorded as BFTBs. All these stinkbugs are members of the Pentatomidae family, which constitutes 26.2% of the known BFTBs. Of these, 17 species are from the subfamily Pentatominae, and only two species belong to the subfamily Phyllocephalinae (*Cressona* spp.). Given that Pentatominae represents the most diverse subfamily within Pentatomidae, accounting for 70.4% of its species globally (Schuh & Weirauch, 2020), the higher incidence of BFTBs in this subfamily is likely reflective of its intrinsic species richness. Meanwhile, other diverse families within Pentatomoidea, such as Plataspidae, Cydnidae, Tessaratomidae, Scutelleridae, and Acanthosomatidae, show no records of BFTBs. This absence may be linked to their evolutionary relationships predominantly with trees rather than with Poaceae (Blount et al., 2015; Zheng, 1994).

In the Lygaeoidea superfamily, 5 families, 9 genera with 13 species of BFTBs were recorded. Among them, 8 species belong to Blissidae, 2 species belong to Colobathristidae and the remaining 3 species belong to Heterogastridae, Malcidae, and Berytidae, respectively. Blissidae species typically exhibit a narrow and flat body, predominantly feeding on Poaceae plants (Wang et al., 2022). They are commonly found in the sheaths of reeds (*Dimorphopterus japonicus* (Hidaka, 1959)), wild rice (*Macropes raja* Distant, 1909), sugarcane (*Cavelerius saccharivorus* (Okajima, 1922)) and other Poaceae plants (Gao, 2010; Wang et al., 2022). Consistent with our field observations, Blissidae species inhabit almost exclusively in bamboo sheaths (Slater & Brailovsky, 1983, 1990). In contrast, the two most diverse families within Lygaeoidea, Rhyparochromidae and Lygaeidae, do not have recorded instances of BFTBs. This absence is likely due to their seed‐feeding habits (Ye et al., 2022). Bamboo, being a Poaceae plant, undergoes dieback after flowering and seeding, often resulting in the demise of entire populations (Chai et al., 2006). Moreover, the flowering cycle of bamboo is notably protracted, typically spanning 3–120 years under favorable climatic conditions (Wang, 2009). This infrequency in seed availability on the ground presents significant ecological barriers for Rhyparochromidae and Lygaeidae to adapt to bamboo‐feeding habits.

In the Coreoidea superfamily, 2 families, 12 genera with 31 species of BFTBs were recorded. Specifically, the Coreidae family comprises 6 genera with 19 species, while the Alydidae family includes 6 genera with 12 species. They are the two biggest families in the Coreoidea superfamily, constituting approximately 82.6% and 9.1% of the superfamily respectively (Schuh & Weirauch, 2020). Most BFTBs in Coreidae (except for *Fracastorius cornutus* Distant, 1902, and *Homoeocerus striicornis* Scott, 1874) belong to the tribe of Cloresmini and Manocoreini. All BFTBs in Alydidae belong to the tribe of Micrelytrini. These three tribes represent 96.8% of BFTBs within Coreoidea and 47.7% of all BFTBs in China, indicating their significant adaptation to bamboo. Geographically, Cloresmini is confined to the Oriental Region and New Guinea in the Australian Region (Jiang, 2023); Manocoreini is predominantly found in southern China and is known for its oligophagous bamboo diet (Ren, 1983); Micrelytrini, with 81 species, is primarily located in the Oriental and Neotropical regions (32 and 34 species, respectively), with the remaining species spread across the Palaearctic, Nearctic, and Neotropical regions, absent in the Australasian Region (Yi, 2016). This distribution mirrors the global spread of the Bambusoideae (Shi et al., 2020; Yi et al., 2008). The fact that all species with clear feeding records in these three tribes exhibit bamboo‐feeding habits, along with the correspondence between their distribution and that of their host bamboo, suggests that they may be oligophagous groups associated with bamboo. Additionally, Rhopalidae, as the third‐largest taxon in Coreoidea, has no records of BFTBs. This family is considered a northerly distributed taxon in Eurasia, with only a few species in the subtropical‐tropical region (Zheng, 1994).

### 4.2 Bamboo‐feeding true bugs distribution in China

The spatial diversity patterns of BFTBs in China may be correlated with the distribution of host bamboo. This correlation between insect diversity and host plant distribution is well‐documented across various taxa. For instance, wax scales (Insecta: Hemipetra: Coccidae) feed extensively on more than 200 families of vascular plants, and the spatial diversity pattern of certain families of wax scales is positively correlated with the diversity of host plant family (Lin et al., 2015). Similarly, Du et al. (2020) identified host plant diversity as the primary determinant of diversity among Drepanosiphine aphids (Du et al., 2020). The diversity of the butterfly family Nymphalidae has also been linked to the diversity of their host plants (Janz et al., 2006). Within China, the Bambusoideae subfamily is divided into three subzones based on the geographical markers of the Qinling–Huaihe–Zhongtiao Mountain line and the Tropic of Cancer, with an increasing number of genera from north to south (Liu, Qiu, et al., 2018). Correspondingly, the diversity of BFTBs in China exhibits a gradient from low to high across these subzones, suggesting a strong biogeographical match between BFTBs and their host bamboo.

The spatial diversity pattern of BFTBs could be explained by the water‐energy dynamics hypothesis, and historical climate stability also plays a role in influencing it. Consistent with the Chinese endemic species of four family groups in Heteroptera and Hemiptera species in China, the minimum temperature of coldest month and annual precipitation dominate the diversity distribution of BFTBs (Jiang, Dong, et al., 2022; Li et al., 2021). The water‐energy dynamics hypothesis suggests that greater availability of water and energy provides a robust resource base for biodiversity, allowing more organisms to coexist (O'Brien, 1998, 2006; Wang et al., 2011). This framework has been applied successfully to explain large‐scale spatial diversity patterns across various taxa including vascular plants, mammals, amphibians, and birds globally (Kreft & Jetz, 2007), as well as biodiversity across different altitudes in the Himalayas (Jenkins et al., 2013; Vetaas et al., 2019). In addition, historical climate stability in southern China may supply basic conditions for living of BFTBs (Fick & Hijmans, 2017). The distribution of BFTBs is indirectly influenced by this hypothesis through its impact on the growth and distribution of host bamboo. Temperature and moisture, crucial for bamboo growth, affect seed germination, shoot differentiation, and the renewal growth of underground rhizomes (Wang, 2009). Similarly, moisture influences bamboo shoot emergence and growth, typically requiring annual precipitation of 1000 to 2000 mm (Li et al., 2011). The combined effects of temperature and moisture are critical for bamboo viability (Liu, Qiu, et al., 2018). The similarity in the spatial diversity patterns of BFTBs and the cold tolerance of bamboo further underscores this relationship (Yi et al., 2008). Therefore, the spatial diversity of host bamboo, combined with the water‐energy dynamics hypothesis, maybe the main driving force for the spatial diversity of BFTBs.

A high diversity of BFTBs in a region does not necessarily indicate more severe pest issues. The major bamboo‐producing provinces—Fujian, Jiangxi, Hunan, Zhejiang, Sichuan, Guangdong, Guangxi, and Anhui—comprise approximately 90% of China's bamboo cultivation areas (Feng & Li, 2023). The pest problems also happened frequently in these areas (Xu & Wang, 2004). Despite Xishuangbanna's greater diversity of BFTBs, the fragmented nature of bamboo plantations and the high species diversity within the community may serve as a natural barrier against pest outbreaks (Altieri et al., 1984; Keesing & Ostfeld, 2024; Qi et al., 2022). In addition, our research innovatively applied the MCP‐AT method to simulate distribution areas for species with 2 to 4 recorded occurrences. Previously, point‐to‐grid mothed was usually applied to simulate their distributions (Jiang, Dong, et al., 2022). The MCP‐AT method was inspired by plant distribution research. Due to the sensitivity of plant distribution to elevation, researchers have successfully simulated plant distribution areas by integrating county locations with elevation data (Zhang et al., 2015). The MCP‐AT method focuses on only one environmental factor (AT), neglecting others like precipitation, which also impacts insect distribution. While incorporating numerous environmental variables could lead to overly conservative predictions, thus limiting the model's applicability, the MCP‐AT method effectively refines the potential distribution areas for species with limited occurrence records, offering a more targeted approach for ecological modeling.

### 4.3 The insufficient research

Our research did not confirm the feeding habits of insects through molecular methods. The ideal approach to confirm the feeding habit would involve obtaining the bamboo DNA from the intestinal food debris. However, despite sequencing approximately 30 samples in pre‐experiment, we were unable to detect any plant DNA. The deeply fragmented food caused by piercing‐sucking mouthparts may contribute to this failure. Furthermore, although Rhyparochromidae and Cydnidae had no records of BFTBs in our research, they still may potentially feed on bamboo seeds. Our limited encounters with bamboo blossoms and seeds hindered the collection efforts for Rhyparochromidae. Our result has primarily focused on BFTBs inhabiting bamboo canopies and shoots, neglecting ground‐dwelling species. Groups such as Cydnidae and Rhyparochromidae which mainly feed on plant roots or seeds, require more intensive collection efforts in the future. We will further apply more collecting methods to enrich the records.

## AUTHOR CONTRIBUTIONS

**Kun Jiang:** Conceptualization (equal); formal analysis (lead); investigation (lead); methodology (lead); software (equal); visualization (lead); writing – original draft (lead). **Juhong Chen:** Formal analysis (supporting); investigation (supporting); supervision (supporting); visualization (supporting); writing – review and editing (supporting). **Shujing Wang:** Investigation (supporting). **Yanfei Li:** Investigation (supporting). **Danli Zhang:** Supervision (supporting). **Haoyuan Hu:** Supervision (supporting). **Wenjun Bu:** Conceptualization (equal); data curation (lead); funding acquisition (lead); project administration (lead); resources (equal); supervision (lead); writing – review and editing (lead).

## CONFLICT OF INTEREST STATEMENT

The authors declare that there are no conflicts of interest.

## Supporting information


**Data S1:** Supporting Information.

## Data Availability

All code and datasets needed to fully reproduce our analyses can be found in the manuscript's supporting information and Dryad: DOI: 10.5061/dryad.x95x69prw.
