# Supplementary material for: Diversity and distribution of bamboo‐feeding true bugs in China
Source: Ecol Evol. 2024 Jul 18;14(7):e11563. doi: 10.1002/ece3.11563 (PMC11255406; doi:10.1002/ece3.11563)
Supplement: Supplementary file 1 — Data S1: Supporting Information. [file ECE3-14-e11563-s001.docx]

**Table S1.** Bamboo-feeding true bugs in China

| Species | Reference |
| --- | --- |
| **Miridae (3 genera 4 species)** |  |
| *Mecistoscelis scirteloides* Reuter, 1891 | (Zheng, 1994) |
| *Mystilus priamus* Distant, 1904 | (Zheng, 1994) |
| *Elthemidea picea* Zheng, 1992 | (Zheng, 1994) |
| *E. sichuanese* Zheng, 1992 | (Zheng, 1994) |
| **Blissdae (5 genera 8 species)** |  |
| *Pirkimerus japonicus* (Hidaka,1961) | (Zheng, 1994) |
| *Macropes robustus* Zheng & Zou, 1982 | (Xu et al., 2004; Zheng, 1994) |
| *M. harringtonae* Slater, Ashlock & Wilcox, 1969 | (Zheng, 1994) |
| *M. maai* Slater & Wilcox, 1973 | (Zheng, 1994) |
| *Iphicrates spinicaput* Scott, 1874 | (Zheng, 1994) |
| *I*. *weni* Zheng, 1986 | (Zheng, 1994) |
| *Dimorphopterus japonicus* (Hidaka, 1959) | (Zheng, 1994) |
| **Colobathristidae (1 genus 2 species)** |  |
| *Phenacantha viridipennis* Horvath, 1904 | (Zheng, 1994) |
| *P. bicolor* (Distant,1901) | (Zheng, 1994) |
| **Alydidae (6 genera 12 species)** |  |
| *Acestra sinica* Dallas 1852 | (Zheng, 1994) |
| *Distachys vulgaris* Hsiao, 1964 | (Zheng, 1994) |
| *Marcius longirostris* Hsiao, 1964 | (Zheng, 1994) |
| *Tuberculiformia subinermis* Ahmad, 1967 | (Zheng, 1994) |
| **Coreidae (6 genera 20 species)** |  |
| *Notobitus excellens* Distant, 1879 | (Zheng, 1994) |
| *N. sexguttatus* (Westwood, 1842) | (Zheng, 1994) |
| *N. elongatus* Hsiao, 1977 | (Zheng, 1994) |
| *N. meleagris* (Fabricius, 1787) | (Xu, & Wang, 2004; Zheng, 1994) |
| *N. montanus* Hsiao, 1963 | (Xu, & Wang, 2004; Zheng, 1994) |
| *N. fermoralis* Chen, 1986 | (Chen, 1986) |
| *Notobitiella elegans* Hsiao, 1963 | (Zheng, 1994) |
| *N. bispina* Jiang, Chen et Bu, 2022 | (Jiang et al., 2022) |
| *Cloresmus yunnanensis* Hsiao, 1963 | (Zheng, 1994) |
| *C.* *pulchellus* Hsiao, 1963 | (Zheng, 1994) |
| *C. modestus* Distant, 1901 | (Zheng, 1994) |
| *C. similis* (Dallas, 1852) | (Hsiao, 1977) |
| *Manocoreus marginatus* Hsiao, 1964 | (Ren, 1983) |
| *M. yunnanensis* Hsiao, 1964 | (Ren, 1983) |
| *M. vulgaris* Hsiao, 1964 | (Ren, 1983; Zheng, 1994) |
| *M. montanus* Hsiao, 1964 | (Ren, 1983) |
| *M. astinus* Ren, 1983 | (Ren, 1983) |
| *Fracastorius cornutus* Distant, 1902 | (Ren, 1983) |
| *Homoeocerus striicornis* Scott, 1874 | (Xu, & Wang, 2004) |
| **Pentatomidae (11 genera 20 species)** |  |
| *Cressona valida* Dallas, 1851 | (Zheng, 1994) |
| *C. divaricata* Zheng & Zou, 1982 | (Zheng, 1994) |
| *Vitruvius insignis* Distant, 1901 | (Zheng, 1994) |
| *Aenaria pinchii* Yang, 1934 | (Chen, 1989; Zhang, 1995; Zheng, 1994) |
| *A. lewisi* (Scott,1874) | (Chen, 1989) |
| *A. bivitta* Fan & Liu, 2009 | (Fan et al., 2009) |
| *A.* *zhangi* Chen, 1989 | (Zhang, 1995) |
| *Halyabbas unicolor* Distant, 1900 | (Zheng, 1994) |
| *Brachymna tenuis* Stål, 1861 | (Xu, & Wang, 2004; Zheng, 1994) |
| *B. bificeps* Chen, 2000 | (Zhang, 1995) |
| *B. humerata* Chen, 1989 | (Chen, 1989) |
| *Zouicoris elegans* Zheng,1986 | (Zheng, 1994) |
| *Critheus lineatifrons* Stål, 1870 | (Zheng, 1994) |
| *C. indicus* (Distant, 1900) | (Zheng, 1994) |
| *Hippotiscus dorsalis* (Stål, 1869) | (Xu, & Wang, 2004; Zheng, 1994) |
| *Paterculus parvus* Hsiao & Cheng, 1977 | (Zheng, 1994) |
| *P.elatus* (Yang, 1934） | (Zheng, 1994) |

**Table S1.** Environmental factors used in analyis of spatial diversity pattern driving forces

| Categories | Abbreviations | Environment factors |
| --- | --- | --- |
| Temperature | AMT | Annual mean temperature |
|  | MTWM | Max temperature of warmest month |
|  | MTCM | Min temperature of coldest month |
| Precipitation | AP | Annual precipitation |
|  | PWM | Precipitation of wettest month |
|  | PDM | Precipitation of driest month |
| Habitat diversity | ELE | Elevation |
|  | ROU | Roughness |
|  | ER | Elevation range |
| Historical climate change | PANO | Anomaly of AP between the Last Glacial Maximum and present |
|  | TANO | Anomaly of AMT between the Last Glacial Maximum and present |
| Human interference | POP | Population Density |
|  | BUS | Built-up Surface |

**Table S2.** Newly records of bamboo-feeding true bugs in China

| Species | Sample number |
| --- | --- |
| **Alydidae (4 genera 8 species)** |  |
| 1. *Anacestra malayana* Distant, 1903 | 159 |
| 2. *Anacestra hirticornis* Hsiao, 1964 | 53 |
| 3. *A. spiniger* Hsiao, 1965 | 25 |
| 4. *Anacestra* sp. | 7 |
| 5. *Marcius nigrospinosus* Ren, 1993 | 8 |
| 6. *M. sichuananus* Ren, 1993 | 6 |
| 7. *Marcius* sp. | 2 |
| 8. *Paramarcius puncticeps* Hsiao, 1964 | 24 |
| **Blissdae (1 genus 1 species)** |  |
| 9. *Bochrus foveatus* Distant,1879 | 10 |
| **Heterogastridae (1 genus 1 species)** |  |
| 10. *Artemidorus pressus* Distant, 1903 | 10 |
| **Malcidae (1 genus 1 species)** |  |
| 11. *Malcus setosus* Stys, 1967 | 15 |
| **Berytidae (1 genus 1 species)** |  |
| 12. *Yemmalysus parallelus* Štusák, 1972 | 103 |
| **Coreidae (2 genera 3 species)** |  |
| 13. *Cloresmus* sp1 | 2 |
| 14. *Cloresmus* sp2 | 12 |
| **Pentatomidae (3 genera 3 species)** |  |
| 15. *Dabessus albovittatus* Hsiao & Cheng | 3 |
| 16. *Dunnius minor* Zheng & Liu, 1987 | 69 |
| 17. *Paterculus aberrans* Distant, 1921 | 7 |


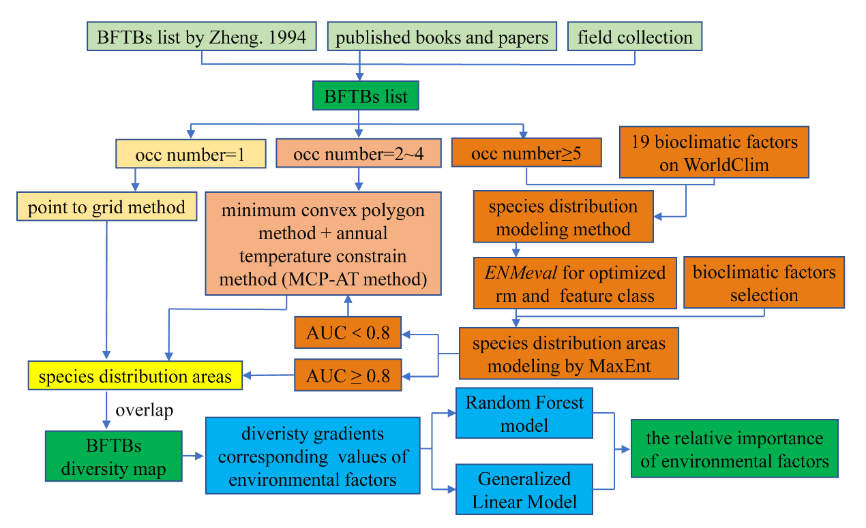


**Figure S1** Flowchart illustrating the main data operation process.

**Table S3** Bamboo-feeding true bugs in the world (except China)

| Species | Country | Reference |
| --- | --- | --- |
| **Pentatomidae (6 genus 7 species)** | | |
| *Piezodorus guildinii* (Westwood, 1837) | America | (Zerbino, Altier et al. 2015) |
| *Placocoris viridis* Mayr.Body, 1864 | Brazil | (Thomas 2021) |
| *Ochrophora montana* (Distant) | India | (Thakur and Firake 2012) |
| *Oregma bambusae* Buckton, 1893 | India | (Choudhury, Akhtar et al. 2007) |
| *Neoadoxoplatys thomasi* Cervantes & Ortega 2014 | Mexico and Venezuela | (Cervantes-Peredo and Ortega-León 2014) |
| *N. saileri* Kormilev 1956 | Kormilev Mexico and Venezuela | (Cervantes-Peredo and Ortega-León 2014) |
| *Brachymna tenuis* Stål, 1861 | South Korea | (Ahn, Kim et al. 2020) |
| **Miridae (3 genus 6 species)** | | |
| *Azumamiris vernalis* Yasunaga 2010 | Central Honshu, Japan | (Yasunaga 2010) |
| *Mecistoscelis scirtetoides* Reuter, 1891 | Myanmar | (Kim, Lim et al. 2021) |
| *M. phillipinensis* Lansbury, 1963 | Myanmar | (Kim, Lim et al. 2021) |
| *M. nigrosignatus* Poppius, 1911 | Myanmar | (Kim, Lim et al. 2021) |
| *M. lansburyi Kim,* Roca-Cusachs, Pham & Jung, 2018 | Myanmar | (Kim, Lim et al. 2021) |
| *Erimiris tenuicornis* Miyamoto & Hasegawa, 1967 | Ecuador | (Yasunaga and Duwal 2017) |
| **Tingidae (1 genus 1 species)** | | |
| *Leptodictya* spp. | South America | (Drake and Ruhoff, 1965) |
| **Lygaeidae (2 genus 2 species)** | | |
| *Neortholomus jamaicensis* Dallas, 1852 | North America | (Ruiz-Sanchez, Peredo et al. 2017) |
| *Cretamystilus herczeki* Kim and Jung 2021 | Myanmar | (Kim, Lim et al. 2021) |
| **Blissinae (11 genus 34 species)** | | |
| *Tympanoblissus ecuatorianus* Dellapé & Minghetti, 2020 | Ecuador | (Minghetti, Braun et al. 2020) |
| *Praetorblissus mexicanus* Cervantes & Brailovsky, 2013 | Mexico | (Nakase and Kato 2011) |
| *Ischnodemus sallei* (Signoret, 1857) | Mexico | (Báez Santacruz, Brailovsky Alperowitz et al. 2017) |
| *I. fulvipes* (De Geer, 1773) | Mexico | (Slater, 1976) |
| *Dimorphopterus japonicus* (Hidaka, 1959) | Japan, South Korea | (Sakata, Kobayashi et al. 2020) |
| *Toonglasa munda* (Slater & Wilcox, 1966) | Mexico | (Báez Santacruz, Brailovsky Alperowitz et al. 2017) |
| *T. tumorosis* (Slater & Wilcox, 1966) | Mexico | (Báez Santacruz, Brailovsky Alperowitz et al. 2017) |
| *T. forficuloides* Distant, 1983 | Mexico | (Slater and Brailovsky, 1983, Slater and Brailovsky, 1990) |
| *T. yushaniacola* Slater & Brailovsky, 1983 | Mexico | (Slater and Brailovsky, 1983, Slater and Brailovsky, 1990) |
| *T. barrerai* Slater & Brailovsky, 1983 | Mexico | (Slater and Brailovsky, 1983, Slater and Brailovsky, 1990) |
| *T. thackstonae* Slater & Brailovsky, 1983 | Mexico | (Slater and Brailovsky, 1983, Slater and Brailovsky, 1990) |
| *T. prunimunda* Slater & Brailovsky, 1990 | Mexico | (Slater and Brailovsky, 1983, Slater and Brailovsky, 1990) |
| *T. elegans* Slater & Brailovsky, 1990 | Mexico | (Slater and Brailovsky, 1983, Slater and Brailovsky, 1990) |
| *T. tumorosoides* Slater & Brailovsky, 1990 | Mexico | (Slater and Brailovsky, 1983, Slater and Brailovsky, 1990) |
| *T. wilcoxae* Slater & Brailovsky, 1990 | Mexico | (Slater and Brailovsky, 1983, Slater and Brailovsky, 1990) |
| *T. tylosis* (Slater & Wilcox, 1966) | Mexico | (Slater and Brailovsky, 1983, Slater and Brailovsky, 1990) |
| *T. umbrata* (Distant, 1893) | Mexico | (Slater and Brailovsky, 1983, Slater and Brailovsky, 1990) |
| *Macropes obnubilus* (Distant, 1883) | Japan | (Nakase and Kato 2011) |
| *M. barringtonae* Slater，Ashlock & Wilcox 1969 | Oriental realm | (Slater, 1976) |
| *M. privus* Distant 1909 | Oriental realm | (Slater, 1976) |
| *M. punctatus* (Walker, 1872) | Oriental realm | (Slater, 1976) |
| *M. spinimanus* Motschulsky, 1859 | Oriental realm | (Slater, 1976) |
| *M. subauratus* Distant, 1904 | Oriental realm | (Slater, 1976) |
| *M. varipennis* (Walker, 1872) | Oriental realm | (Slater, 1976) |
| *Micaredemus eleganoides* Slater, 1967 | Oriental realm | (Slater, 1976) |
| *M. elegans* Slater, 1967 | Oriental realm | (Slater, 1976) |
| *Pirkimerus esakii* Miyamoto & Hidaka, 1960 | Oriental realm | (Slater, 1976) |
| *P. japonicus* (Hidaka, 1961) | Oriental realm | (Slater, 1976) |
| *Riggiella vianai* Kormilev, 1949 | Argentina | (Slater, 1976) |
| *Cavelerius saccharivorus* (Okajima, 1922) | Oriental realm | (Slater, 1976) |
| *Caveloblissus americanus* Slater & Wilcox, 1968 | Oriental realm | (Slater, 1976) |
| *Iphicrates angulatus* Slater, 1961 | Oriental realm | (Slater, 1976) |
| *I. nigritus* Slater, 1961 | Oriental realm | (Slater, 1976) |
| *I. papuensis* Slater, 1961 | Oriental realm | (Slater, 1976) |
| **Coreidae (1 genus 2 species)** | | |
| *Cervantistellus guerrerensis* Brailovsky & Barrera, 2005 | Mexico | (Brailovsky and Barrera 2005) |
| *C. insolitus* Brailovsky & Barrera, 2005 | Mexico | (Brailovsky and Barrera 2005) |
| **Alydidae (1 genus 2 species)** | | |
| *Distachys unicolor* (Scott, 1874) | South Korea | (Ahn, Kim et al. 2020) |
| *D. vulgaris* Hsiao, 1964 | South Korea | (Ahn, Kim et al. 2020) |


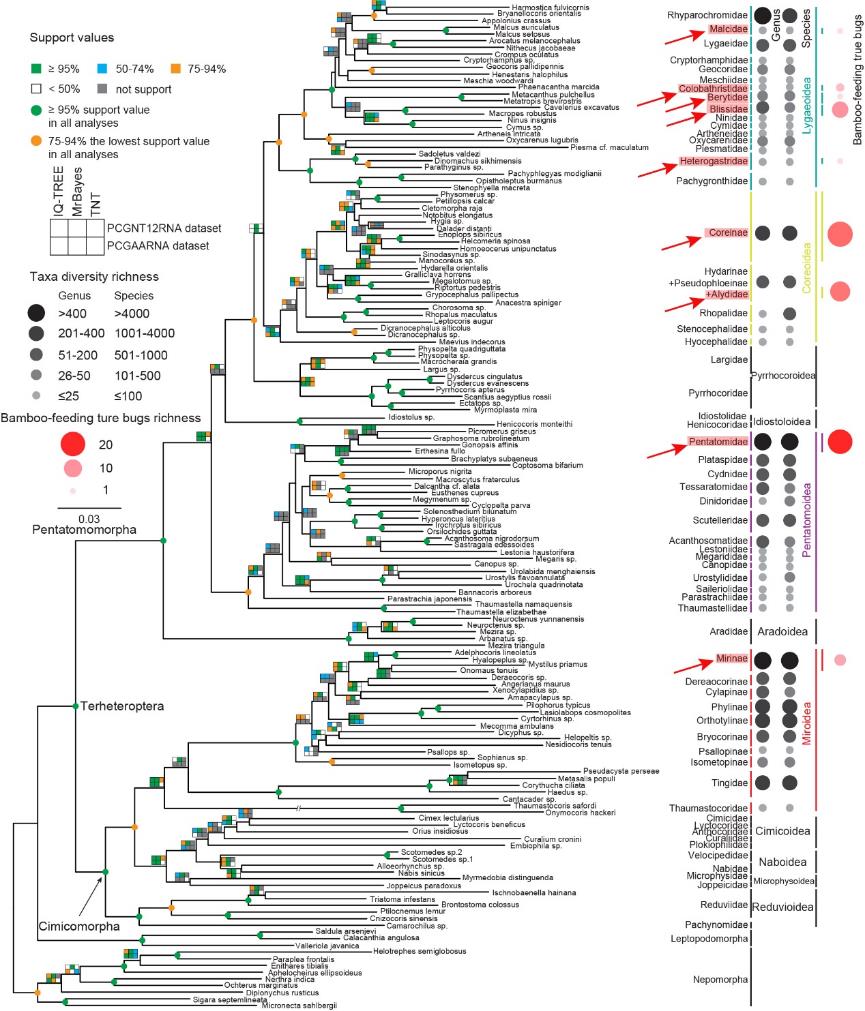


**Figure S2.** Evolutionary position and richness of bamboo-feeding true bugs. Gene tree of phytophagous true bugs from Ye 2023. Red arrow: family or subfamily of bamboo-feeding true bugs.

**Bamboo-feeding true bugs distribution**

ENMeval package was used to evaluate the best model parameters combination. The model parameters were tested by setting different feature class (feature class = L, H, LQ, LQH, LQHP, LQHPT) and regularization multiplier (Regularization multiplier = 0.5, 1, 1.5, 2, 2.5, 3, 3.5, 4). The parameters combination with the lowest delta AICc was selected as the best. Model evaluations for 39 species were as follows.

1. *Acestra malayana*

For *Acestra malayana*, selected environmental factors were BIO02, BIO04, BIO05, BIO09 BIO12, BIO16, BIO19. ENMeval results indicated that the best parameter combination is LQHPT+2 (Figure S2). Under these parameters, the AUC of MaxEnt model is 0.89. Species distribution areas showed on Figure S3.


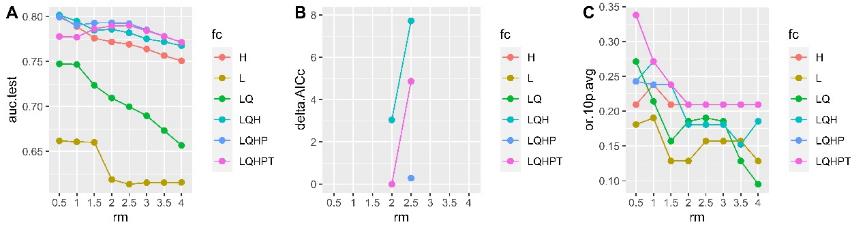


**Figure S3.** ENMeval results for *Acestra malayana*


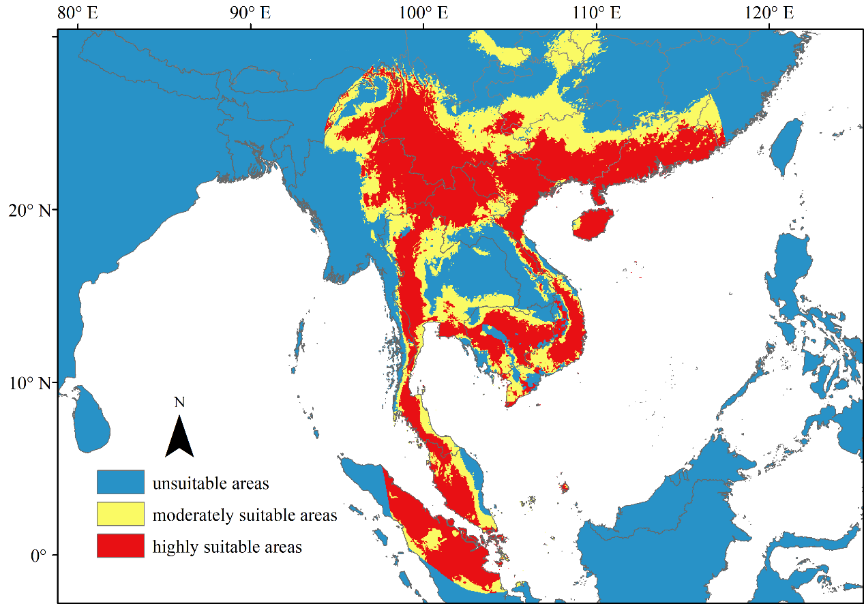


**Figure S4.** The distribution of *Acestra malayana*

2. *Acestra sinica*

For *Acestra sinica*, selected environmental factors were BIO04, BIO05, BIO07, BIO08, BIO09, BIO11, BIO12, BIO16, BIO18. ENMeval results indicated that the best parameter combination is LQH+2.5 (Figure S4). Under these parameters, the AUC of MaxEnt model is 0.88. Species distribution areas showed on Figure S5.


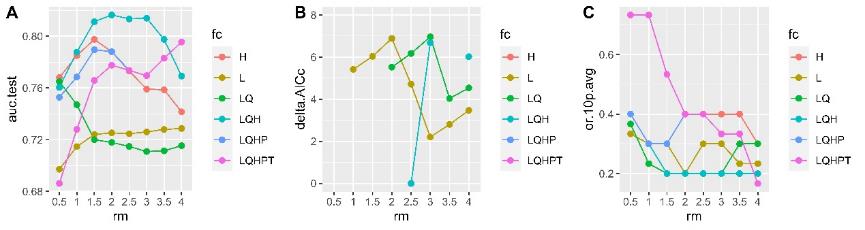


**Figure S5.** ENMeval results for *Acestra sinica*


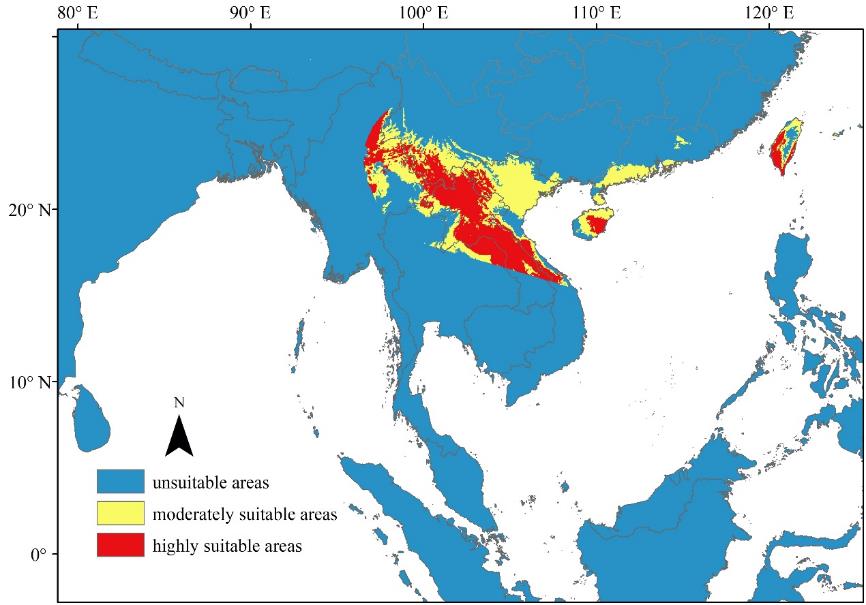


**Figure S6.** The distribution of *Acestra sinica*

3. *Aenaria pinchii*

For *Aenaria pinchii*, selected environmental factors were BIO02, BIO03, BIO05, BIO06, BIO07, BIO08, BIO15, BIO16. ENMeval results indicated that the best parameter combination is LQ+0.5 (Figure S6). Under these parameters, the AUC of MaxEnt model is 0.88. Species distribution areas showed on Figure S7.


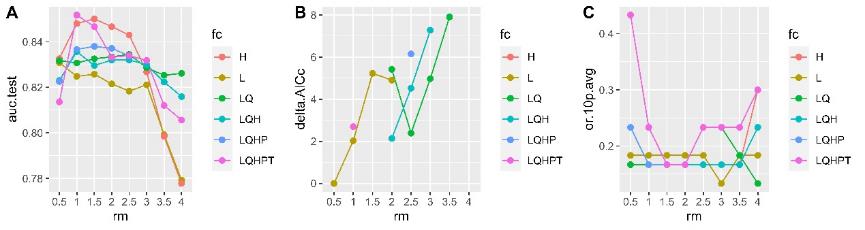


**Figure S7.** ENMeval results for *Aenaria pinchii*


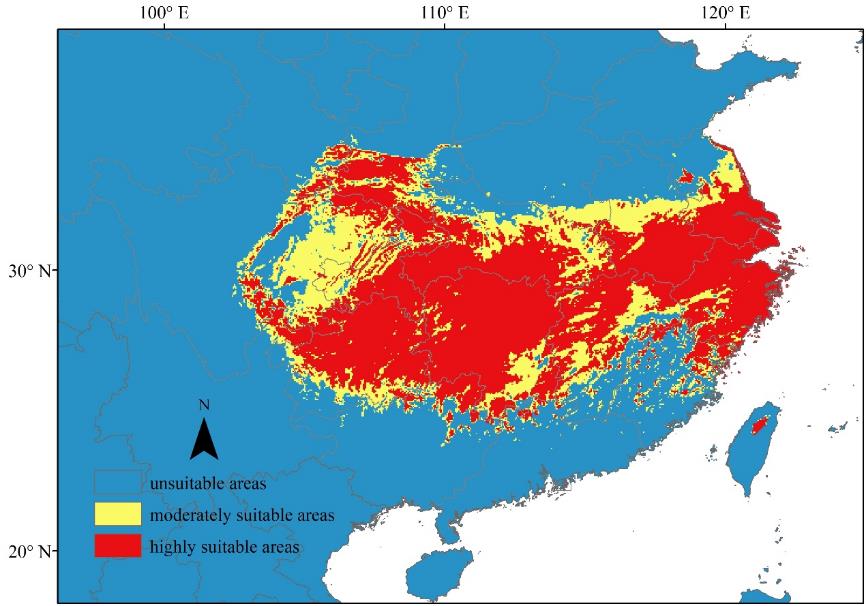


**Figure S8.** The distribution of *Aenaria pinchii*

4. *Aenaria zhangi*

For *Aenaria zhangi*, selected environmental factors were BIO02, BIO05, BIO07, BIO13, BIO14, BIO17. ENMeval results indicated that the best parameter combination is LQHTP+2.5 (Figure S8). Under these parameters, the AUC of MaxEnt model is 0.89. Species distribution areas showed on Figure S9.


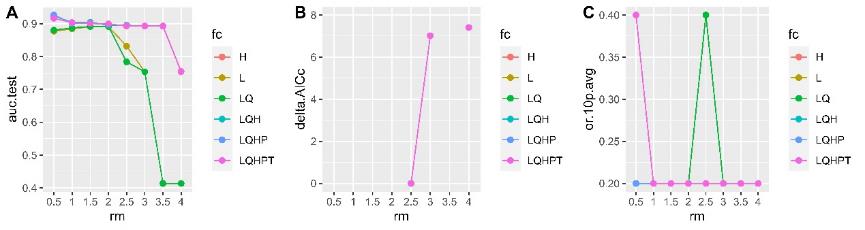


**Figure S9.** ENMeval results for *Aenaria zhangi*


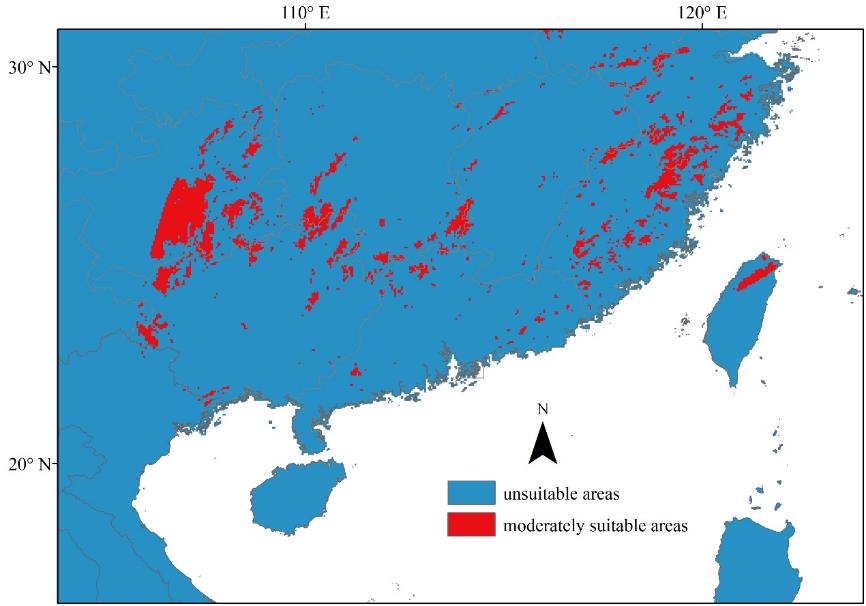


**Figure S10.** The distribution of *Aenaria zhangi*

5. *Anacestra hirticornis*

For *Anacestra hirticornis*, selected environmental factors were BIO01, BIO02, BIO07, BIO08, BIO10, BIO12, BIO14, BIO15, BIO18. ENMeval results indicated that the best parameter combination is LQH+3.5 (Figure S10). Under these parameters, the AUC of MaxEnt model is 0.97. Species distribution areas showed on Figure S11.


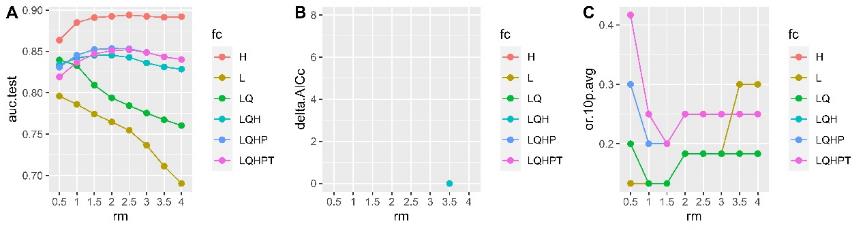


**Figure S11.** ENMeval results for *Anacestra hirticornis*


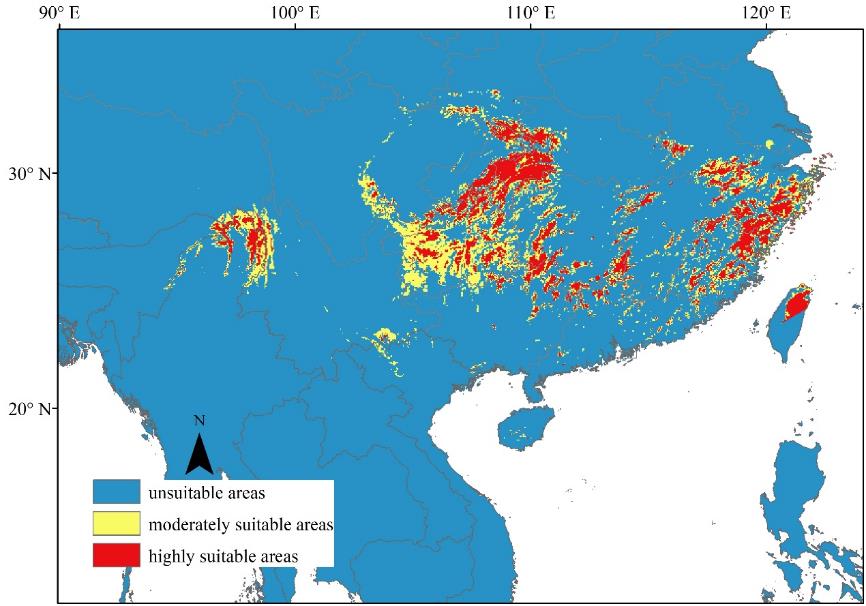


**Figure S12.** The distribution of *Anacestra hirticornis*

6. *Anacestra spiniger*

For *Anacestra spiniger*, selected environmental factors were BIO02, BIO04, BIO05, BIO08, BIO13, BIO14. ENMeval results indicated that the best parameter combination is LQ+1. (Figure S12). Under these parameters, the AUC of MaxEnt model is 0.89. Species distribution areas showed on Figure S13.


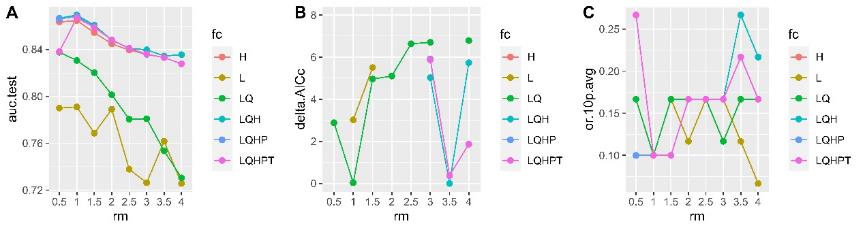


**Figure S13.** ENMeval results for *Anacestra spiniger*


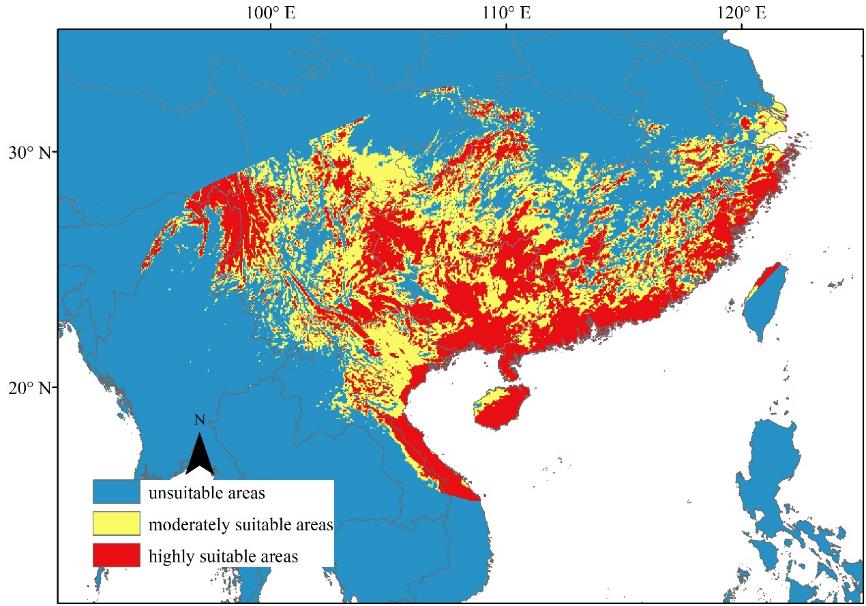


**Figure S14.** The distribution of *Anacestra spiniger*

7. *Artemidorus pressus*

For *Artemidorus pressus*, selected environmental factors were BIO01, BIO02, BIO03, BIO06, BIO07, BIO08, BIO12, BIO15, BIO18, BIO19. ENMeval results indicated that the best parameter combination is L+1.5. (Figure S14). Under these parameters, the AUC of MaxEnt model is 0.73. The model failed to simulated the species distribution, its distribution will simulated by point to grids method.


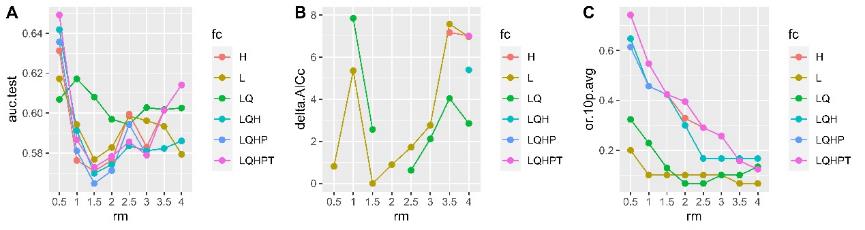


**Figure S15.** ENMeval results for *Artemidorus pressus*

8. *Brachymna tenuis*

For *Brachymna tenuis*, selected environmental factors were BIO02, BIO03, BIO05, BIO08, BIO11, BIO12, BIO15, BIO17. ENMeval results indicated that the best parameter combination is L+0.5(Figure S15). Under these parameters, the AUC of MaxEnt model is 0.85. Species distribution areas showed on Figure S16.


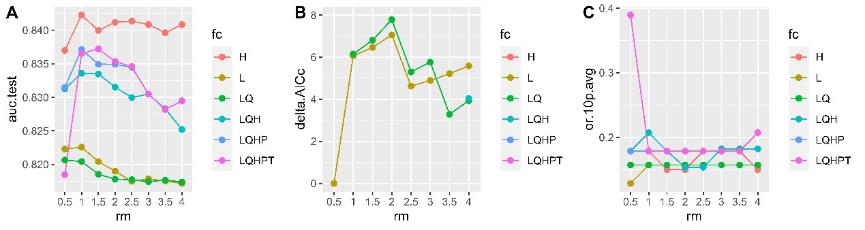


**Figure S16.** ENMeval results for *Brachymna tenuis*


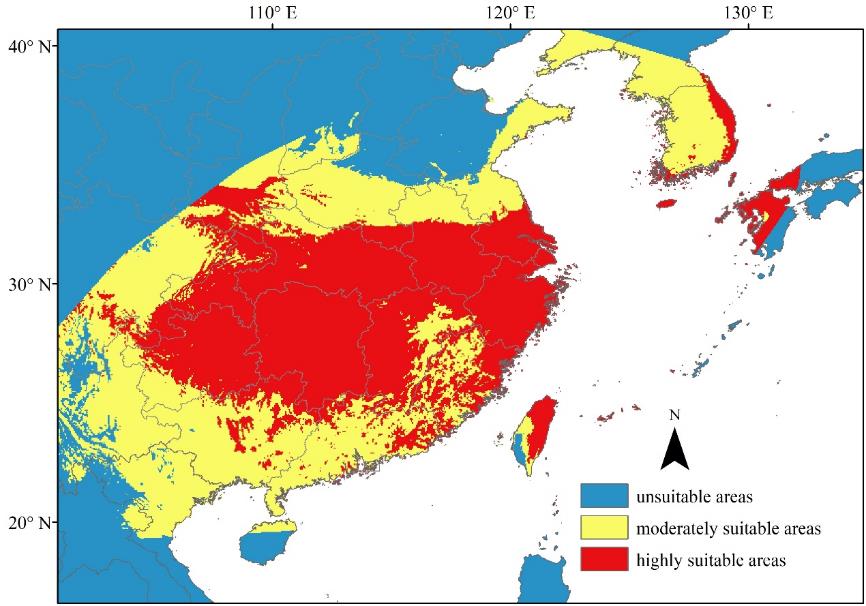


**Figure S17.** The distribution of *Brachymna tenuis*

9. *Cloresmus modestus*

For *Cloresmus modestus*, selected environmental factors were BIO03, BIO07, BIO11, BIO13, BIO14, BIO15, BIO18. ENMeval results indicated that the best parameter combination is LQ+0.5 (Figure S17). Under these parameters, the AUC of MaxEnt model is 0.91. Species distribution areas showed on Figure S18.


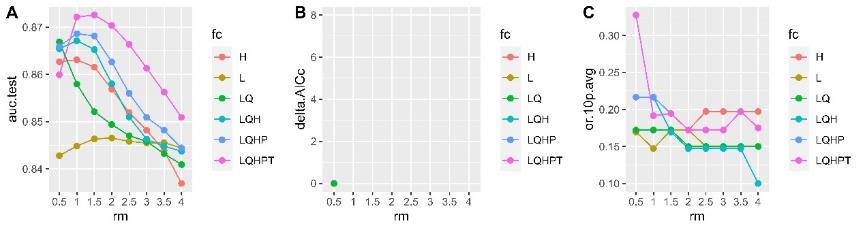


**Figure S18.** ENMeval results for *Cloresmus modestus*


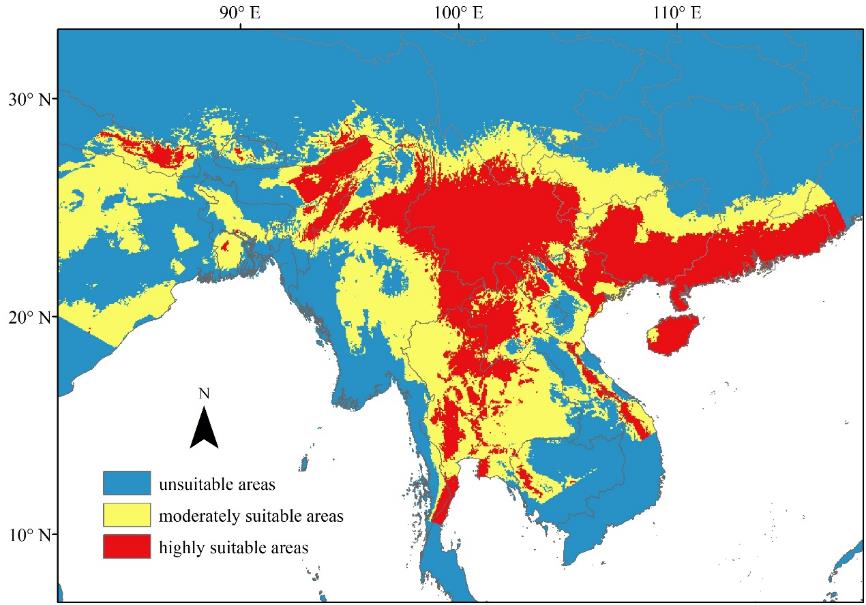


**Figure S19.** The distribution of *Cloresmus modestus*

10. *Cloresmus pulchellus*

For *Cloresmus pulchellus*, selected environmental factors were BIO02, BIO03, BIO06, BIO08, BIO12, BIO14, BIO15. ENMeval results indicated that the best parameter combination is LQ+1.5 (Figure S19). Under these parameters, the AUC of MaxEnt model is 0.91. Species distribution areas showed on Figure S20.


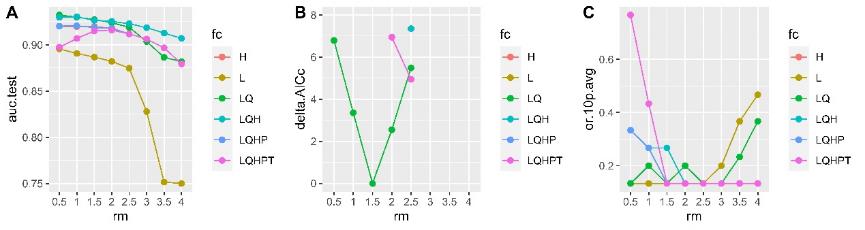
**Figure S20.** ENMeval results for *Cloresmus pulchellus*


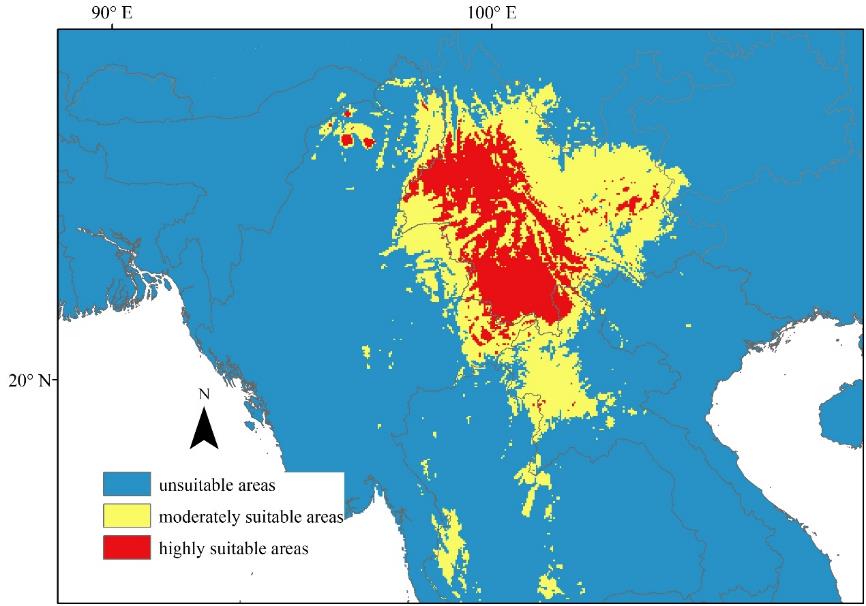


**Figure S21.** The distribution of *Cloresmus pulchellus*

11. *Cloresmus yunnanensis*

For *Cloresmus yunnanensis*, selected environmental factors were BIO03, BIO06, BIO07, BIO11, BIO12, BIO15, BIO18. ENMeval results indicated that the best parameter combination is L+4 (Figure S21). Under these parameters, the AUC of MaxEnt model is 0.69. The model failed to simulated the species distribution, its distribution will simulated by point to grids method.


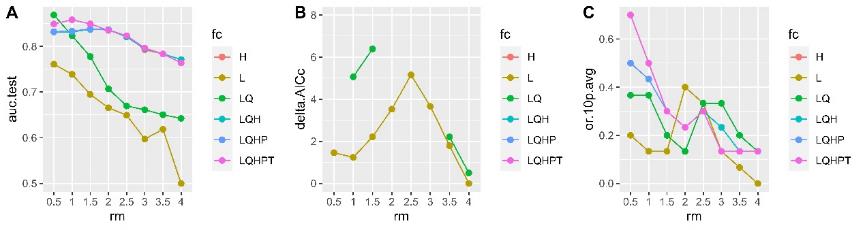


**Figure S22.** ENMeval results for *Cloresmus yunnanensis*

12. *Critheus lineatifrons*

For *Critheus lineatifrons*, selected environmental factors were BIO02, BIO03, BIO07, BIO10, BIO15, BIO16, BIO17. ENMeval results indicated that the best parameter combination is L+2.5 (Figure S22). Under these parameters, the AUC of MaxEnt model is 0.64. The model failed to simulated the species distribution, its distribution will simulated by point to grids method.


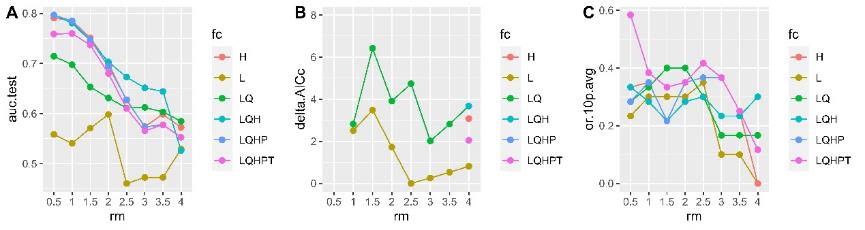


**Figure S23.** ENMeval results for *Critheus lineatifrons*

13. *Dimorphopterus japonicus*

For *Dimorphopterus japonicus*, selected environmental factors were BIO02, BIO03, BIO08, BIO10, BIO11, BIO12, BIO14, BIO18. ENMeval results indicated that the best parameter combination is LQ+0.5 (Figure S23). Under these parameters, the AUC of MaxEnt model is 0.85. Species distribution areas showed on Figure S24.


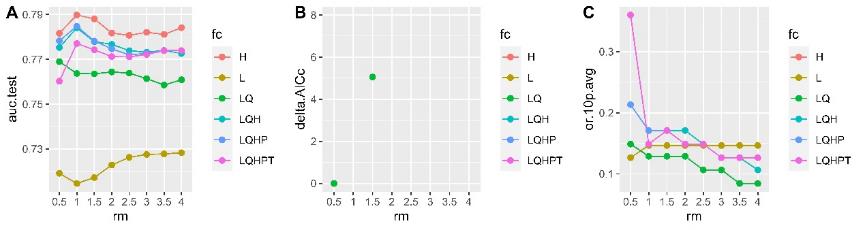


**Figure S24.** ENMeval results for *Dimorphopterus japonicus*


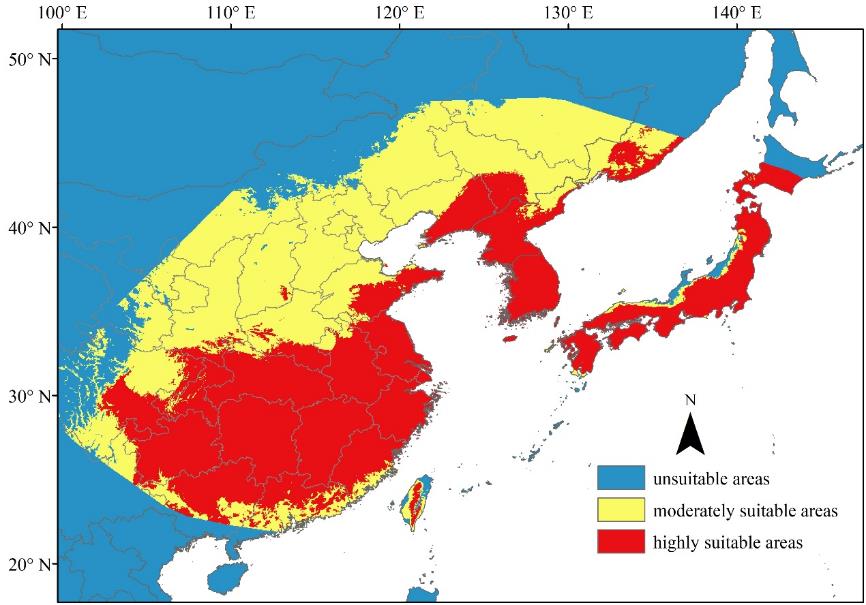


**Figure S25.** The distribution of *Dimorphopterus japonicus*

14. *Distachys vulgaris*

For *Distachys vulgaris*, selected environmental factors were BIO02, BIO07, BIO08, BIO09, BIO10, BIO12, BIO15, BIO17, BIO18. ENMeval results indicated that the best parameter combination is LQ+1 (Figure S25). Under these parameters, the AUC of MaxEnt model is 0.81. Species distribution areas showed on Figure S26.


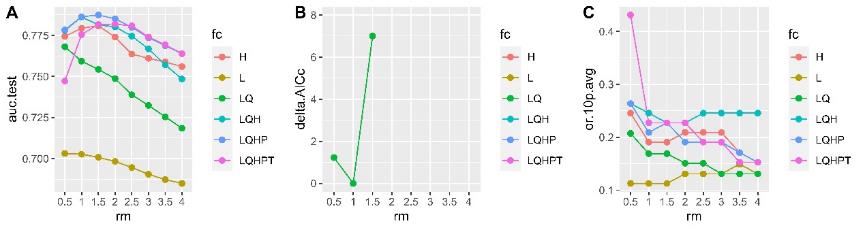


**Figure S26.** ENMeval results for *Distachys vulgaris*


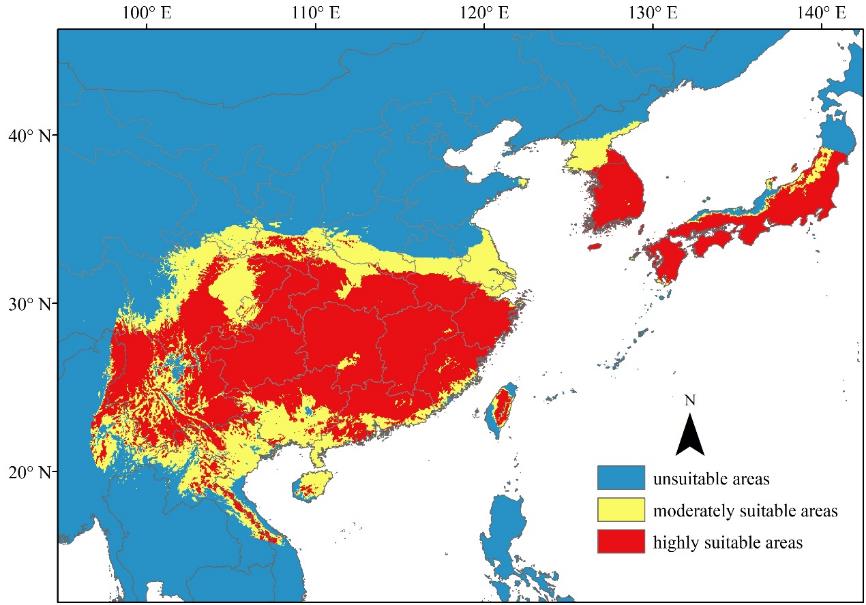


**Figure S27.** The distribution of *Distachys vulgaris*

15. *Dunnius minor*

For *Dunnius minor*, selected environmental factors were BIO02, BIO03, BIO12, BIO13, BIO14, BIO18, BIO19. ENMeval results indicated that the best parameter combination is LQH+3 (Figure S27). Under these parameters, the AUC of MaxEnt model is 0.99. Species distribution areas showed on Figure S28.


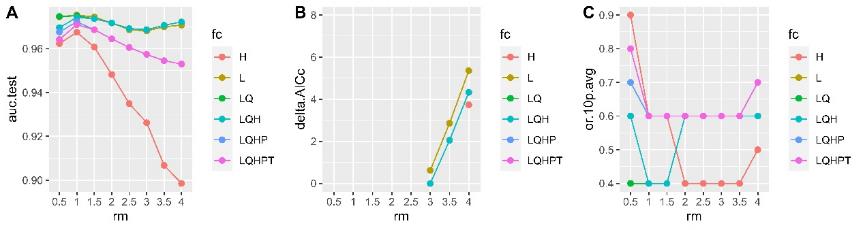


**Figure S28.** ENMeval results for *Dunnius minor*


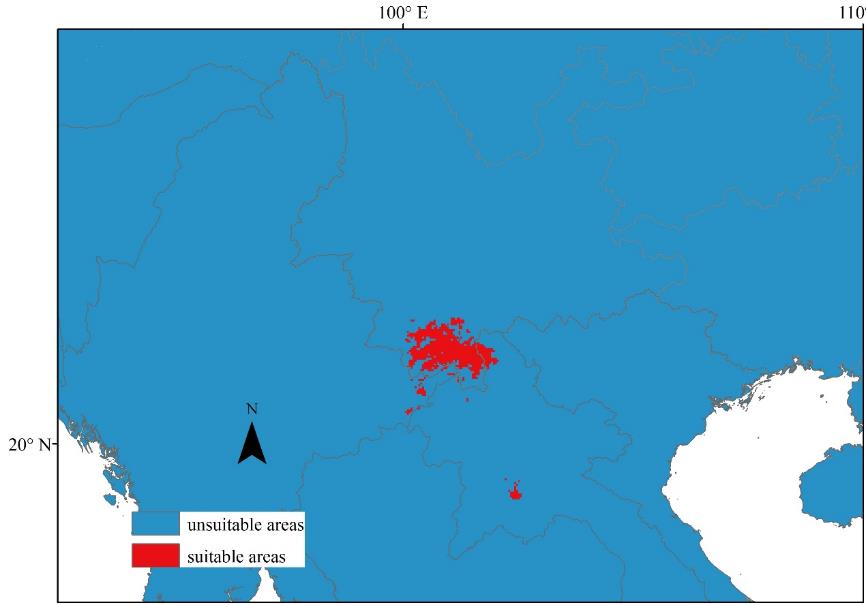


**Figure S29.** The distribution of *Dunnius minor*

16. *Fracastorius cornutus*

For *Fracastorius cornutus*, selected environmental factors were BIO02, BIO04, BIO13, BIO15, BIO17, BIO19. ENMeval results indicated that the best parameter combination is H+3 (Figure S29). Under these parameters, the AUC of MaxEnt model is 0.68. The model failed to simulated the species distribution, its distribution will simulated by point to grids method.
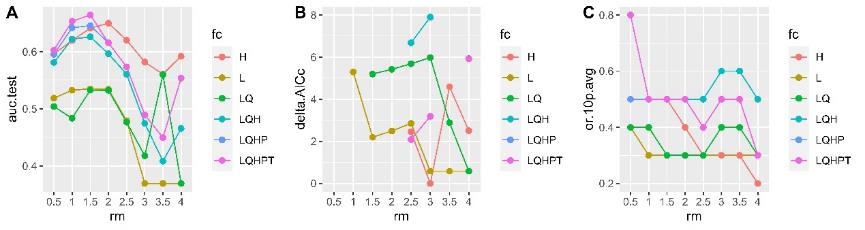


**Figure S30.** ENMeval results for *Fracastorius cornutus*

17. *Halyabbas unicolor*

For *Halyabbas unicolor*, selected environmental factors were BIO02, BIO04, BIO06, BIO10, BIO13, BIO15, BIO18. ENMeval results indicated that the best parameter combination is LQ+0.5 (Figure S30). Under these parameters, the AUC of MaxEnt model is 0.88. Species distribution areas showed on Figure S31.


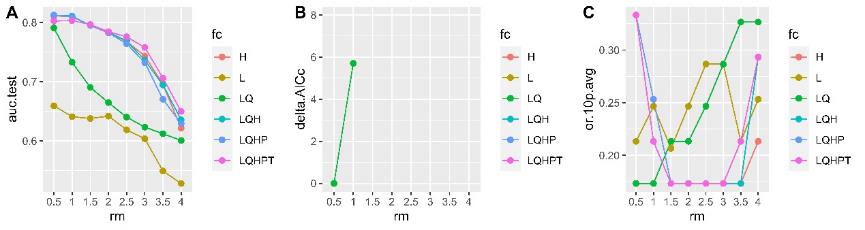


**Figure S31.** ENMeval results for *Halyabbas unicolor*


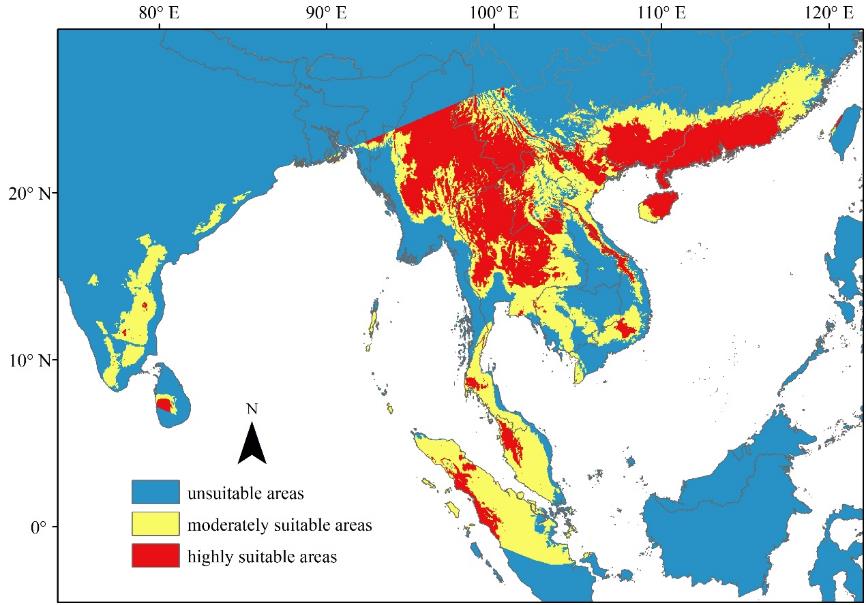


**Figure S32.** The distribution of *Halyabbas unicolor*

18. *Hippotiscus dorsalis*

For *Hippotiscus dorsalis*, selected environmental factors were BIO02, BIO03, BIO04, BIO05, BIO06, BIO08, BIO09, BIO15, BIO16, BIO17. ENMeval results indicated that the best parameter combination is LQHPT+3 (Figure S32). Under these parameters, the AUC of MaxEnt model is 0.84. Species distribution areas showed on Figure S33.


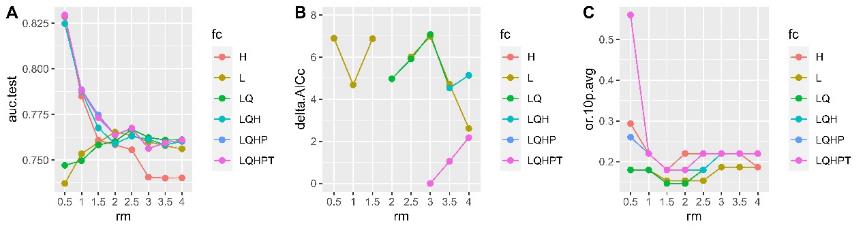


**Figure S33.** ENMeval results for *Hippotiscus dorsalis*


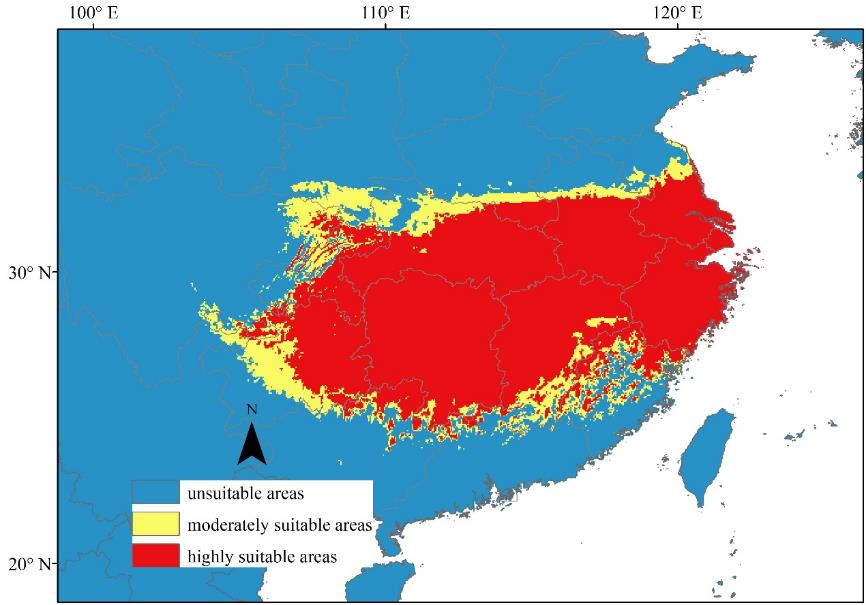


**Figure S34.** The distribution of *Hippotiscus dorsalis*

19. *Homoeocerus striicornis*

For *Homoeocerus striicornis*, selected environmental factors were BIO01, BIO02, BIO03, BIO05, BIO08, BIO12, BIO13, BIO15, BIO18, BIO19. ENMeval results indicated that the best parameter combination is LQHPT+3.5 (Figure S34). Under these parameters, the AUC of MaxEnt model is 0.89. Species distribution areas showed on Figure S35.


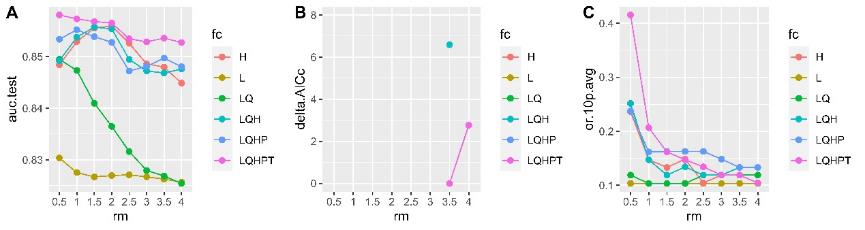


**Figure S35.** ENMeval results for *Homoeocerus striicornis*


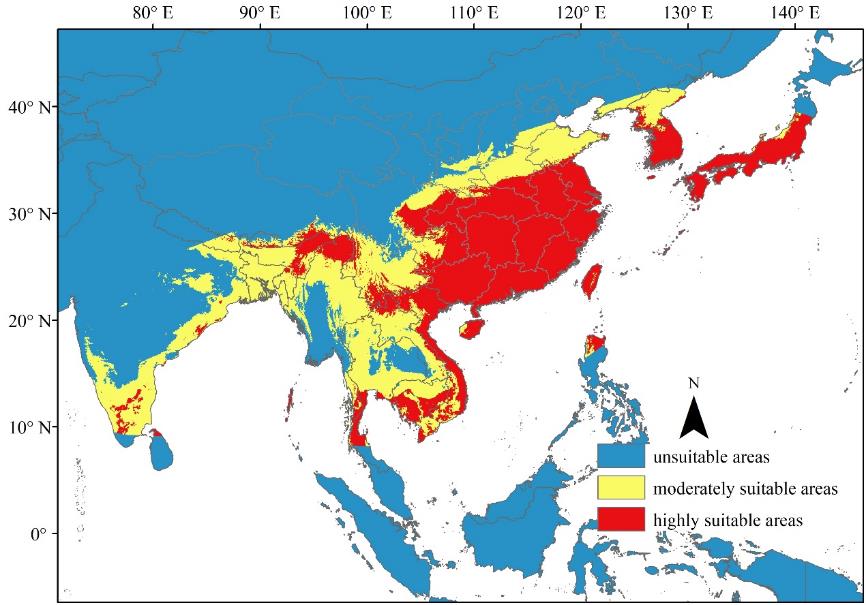


**Figure S36.** The distribution of *Homoeocerus striicornis*

20. *Manocoreus marginatus*

For *Manocoreus marginatus*, selected environmental factors were BIO02, BIO06, BIO07, BIO13, BIO14, BIO16, BIO18, BIO19. ENMeval results indicated that the best parameter combination is L+0.5 (Figure S36). Under these parameters, the AUC of MaxEnt model is 0.92. Species distribution areas showed on Figure S37.


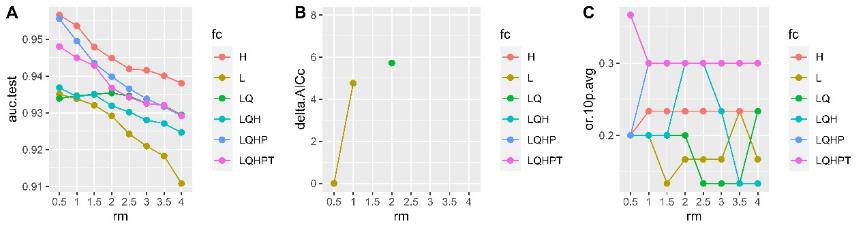


**Figure S37.** ENMeval results for *Manocoreus marginatus*


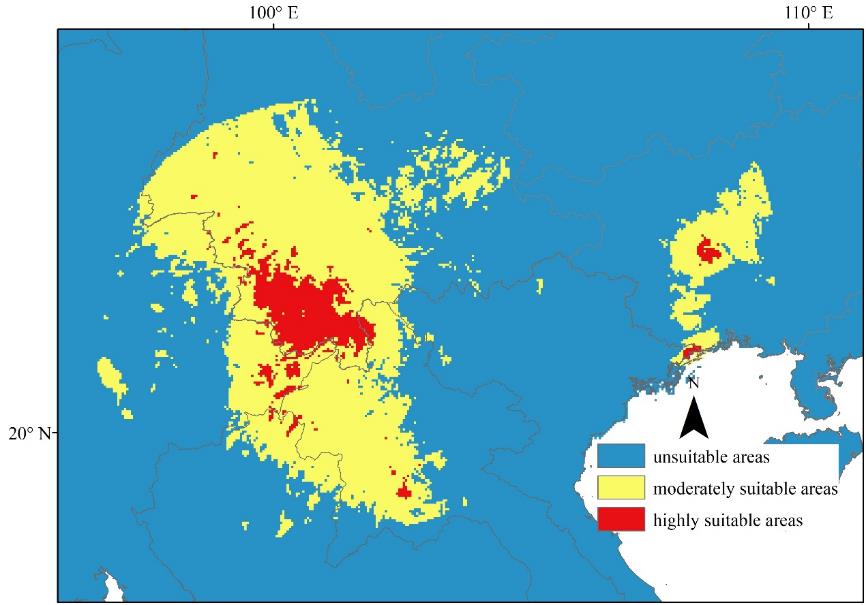


**Figure S38.** The distribution of *Manocoreus marginatus*

21. *Manocoreus vulgaris*

For *Manocoreus vulgaris*, selected environmental factors were BIO02, BIO03, BIO06, BIO07, BIO08, BIO10, BIO12, BIO15, BIO19. ENMeval results indicated that the best parameter combination is LQ+1 (Figure S38). Under these parameters, the AUC of MaxEnt model is 0.95. Species distribution areas showed on Figure S39.


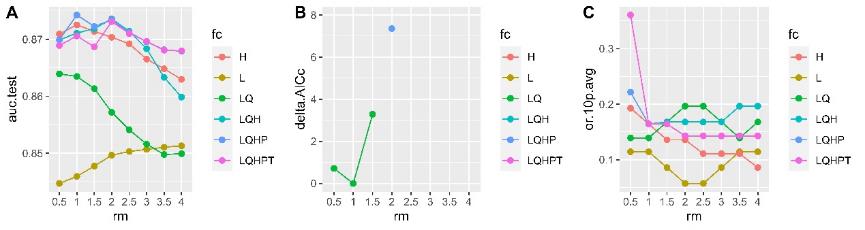


**Figure S39.** ENMeval results for *Manocoreus vulgaris*


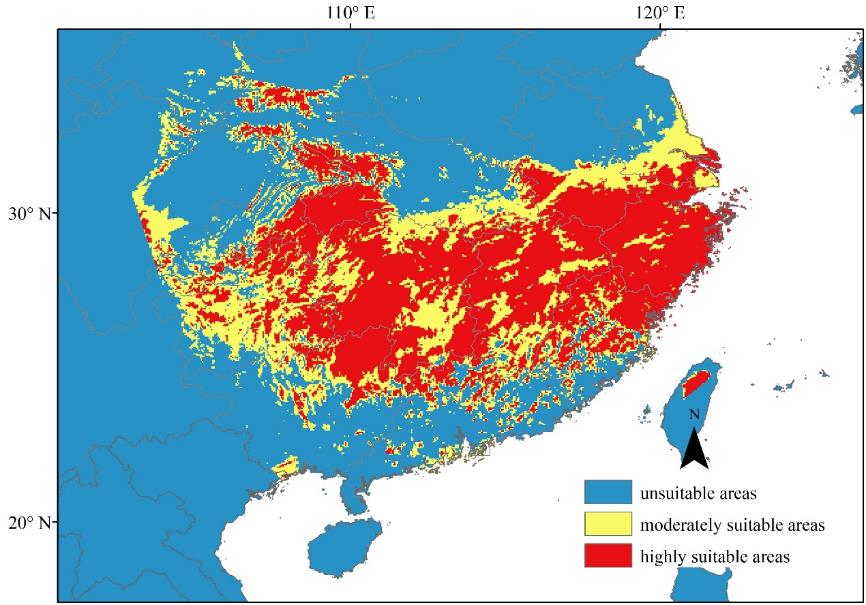
**Figure S40.** The distribution of *Manocoreus vulgaris*

22. *Manocoreus yunnanensis*

For *Manocoreus yunnanensis*, selected environmental factors were BIO03, BIO07, BIO09, BIO10, BIO12, BIO13, BIO18. ENMeval results indicated that the best parameter combination is LQ+2 (Figure S40). Under these parameters, the AUC of MaxEnt model is 0.95. Species distribution areas showed on Figure S41.


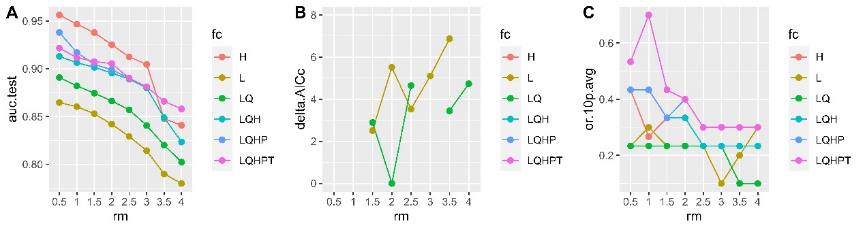


**Figure S41.** ENMeval results for *Manocoreus yunnanensis*


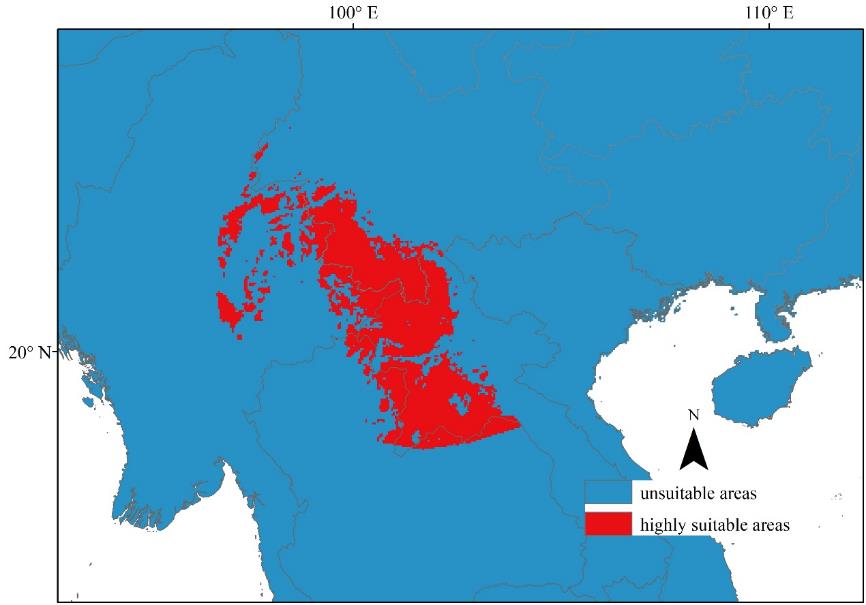


**Figure S42.** The distribution of *Manocoreus yunnanensis*

23. *Macropes harringtonae*

For *Macropes harringtonae*, selected environmental factors were BIO02, BIO03, BIO04, BIO05, BIO08, BIO09, BIO14, BIO15, BIO16. ENMeval results indicated that the best parameter combination is LQ+3.5 (Figure S42). Under these parameters, the AUC of MaxEnt model is 0.79. The model failed to simulated the species distribution, its distribution will simulated by point to grids method.
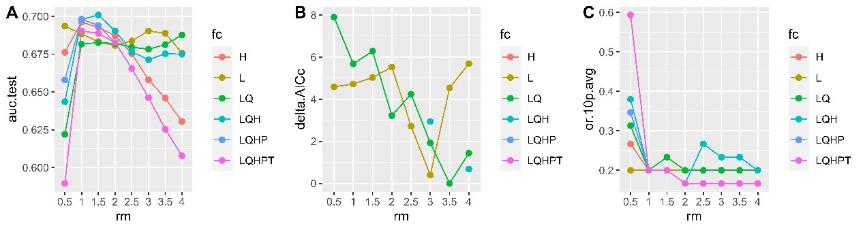


**Figure S43.** ENMeval results for *Macropes harringtonae*

24. *Marcius longirostris*

For *Marcius longirostris*, selected environmental factors were BIO02, BIO03, BIO05, BIO06, BIO08, BIO15, BIO16, BIO17. ENMeval results indicated that the best parameter combination is L+0.5 (Figure S43). Under these parameters, the AUC of MaxEnt model is 0.93. Species distribution areas showed on Figure S44.


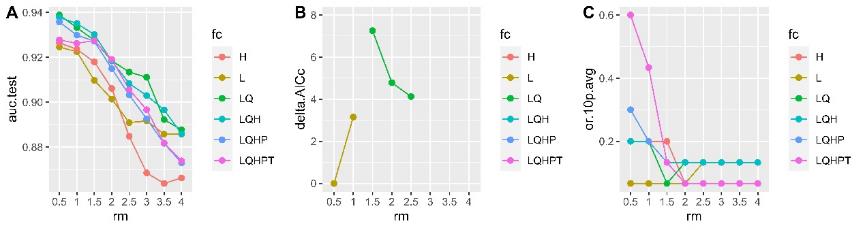


**Figure S44.** ENMeval results for *Marcius longirostris*


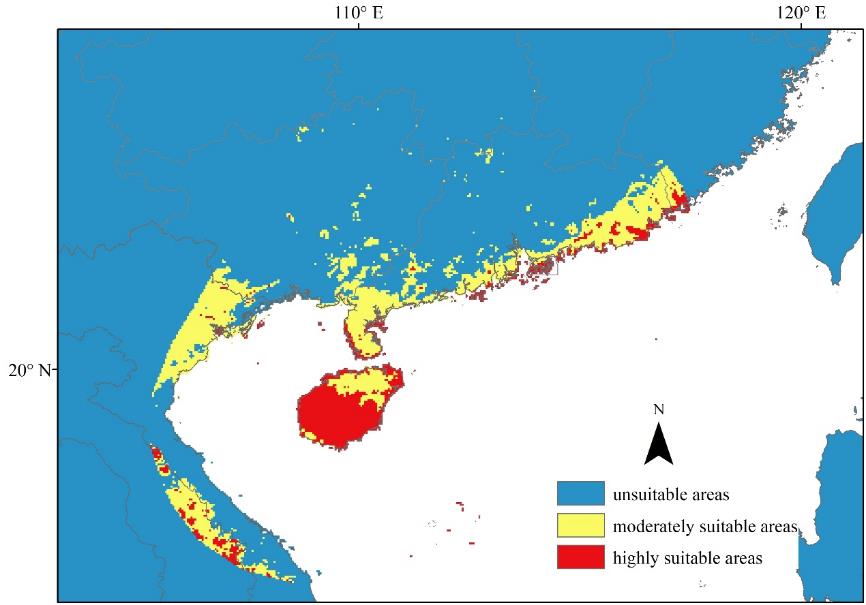


**Figure S45.** The distribution of *Marcius longirostris*

25. *Marcius nigrospinosus*

For *Marcius nigrospinosus*, selected environmental factors were BIO02, BIO03, BIO05, BIO08, BIO09, BIO10, BIO14, BIO15. ENMeval results indicated that the best parameter combination is H+2 (Figure S45). Under these parameters, the AUC of MaxEnt model is 0.99. Species distribution areas showed on Figure S46.


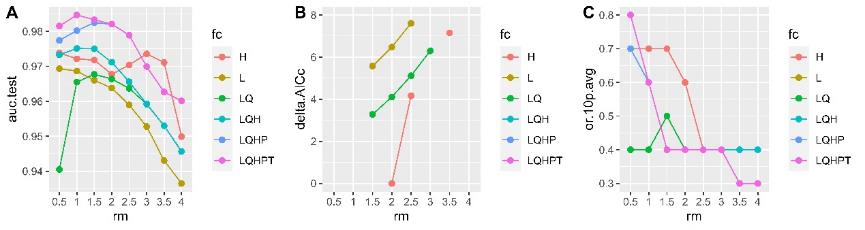


**Figure S46.** ENMeval results for *Marcius nigrospinosus*


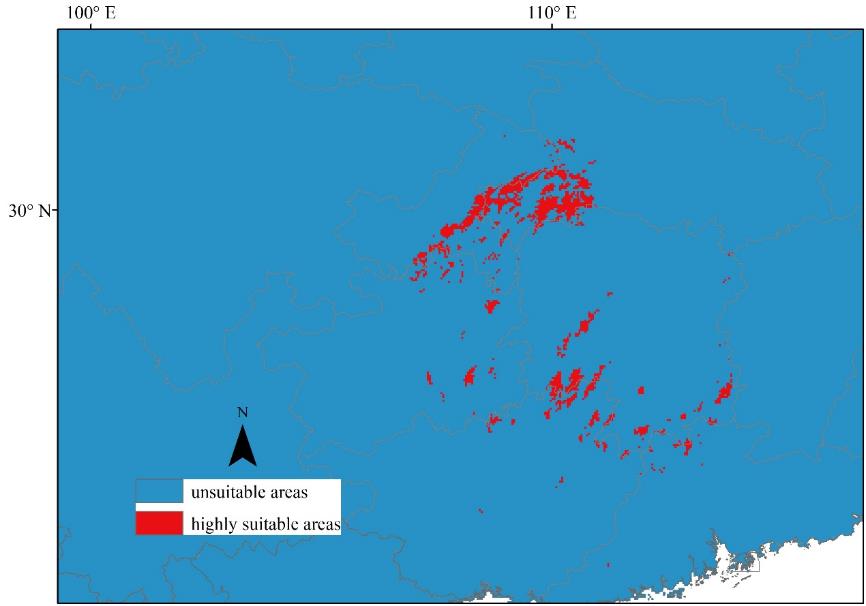


**Figure S47.** The distribution of *Marcius nigrospinosus*

26. *Mecistoscelis scirtetoides*

For *Mecistoscelis scirtetoides*, selected environmental factors were BIO02, BIO04, BIO05, BIO07, BIO11, BIO12, BIO13, BIO17, BIO18. ENMeval results indicated that the best parameter combination is LQ+2 (Figure S47). Under these parameters, the AUC of MaxEnt model is 0.88. Species distribution areas showed on Figure S48.


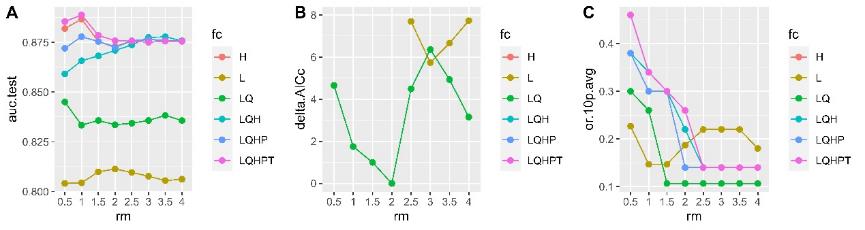


**Figure S48.** ENMeval results for *Mecistoscelis scirtetoides*


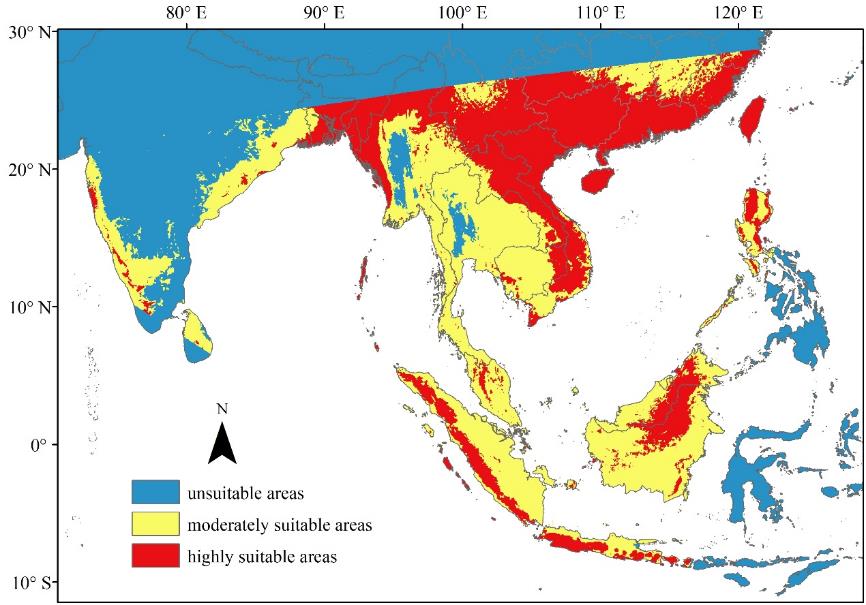


**Figure S49.** The distribution of *Mecistoscelis scirtetoides*

27. *Notobitus elongatus*

For *Notobitus elongatus*, selected environmental factors were BIO04, BIO06, BIO07, BIO13, BIO14, BIO15, BIO17, BIO18. ENMeval results indicated that the best parameter combination is LQ+3 (Figure S49). Under these parameters, the AUC of MaxEnt model is 0.93. Species distribution areas showed on Figure S50.


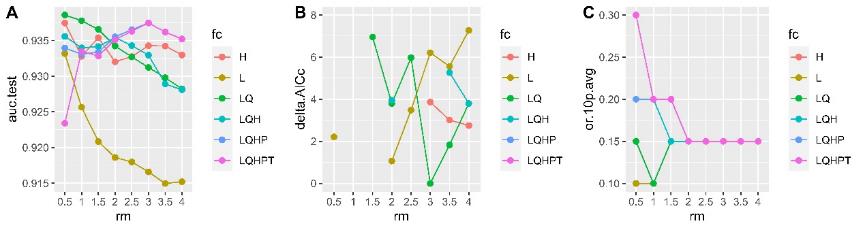


**Figure S50.** ENMeval results for *Notobitus elongatus*


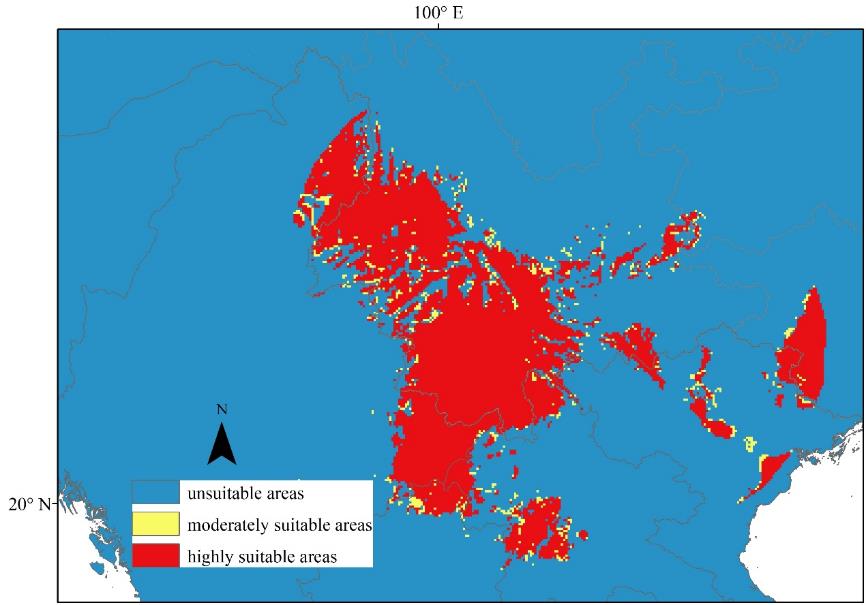


**Figure S51.** The distribution of *Notobitus elongatus*

28. *Notobitus excellens*

For *Notobitus excellens*, selected environmental factors were BIO02, BIO04, BIO06, BIO10, BIO16, BIO17, BIO18. ENMeval results indicated that the best parameter combination is LQH+4 (Figure S51). Under these parameters, the AUC of MaxEnt model is 0.92. Species distribution areas showed on Figure S52.


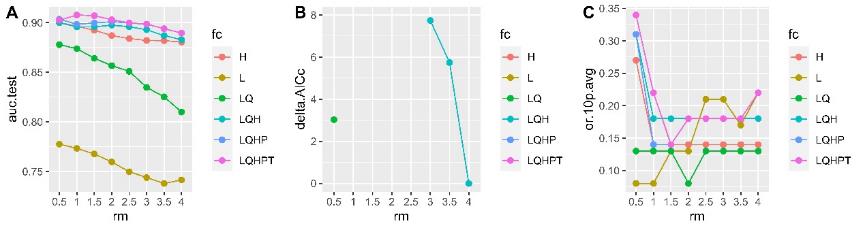


**Figure S52.** ENMeval results for *Notobitus excellens*


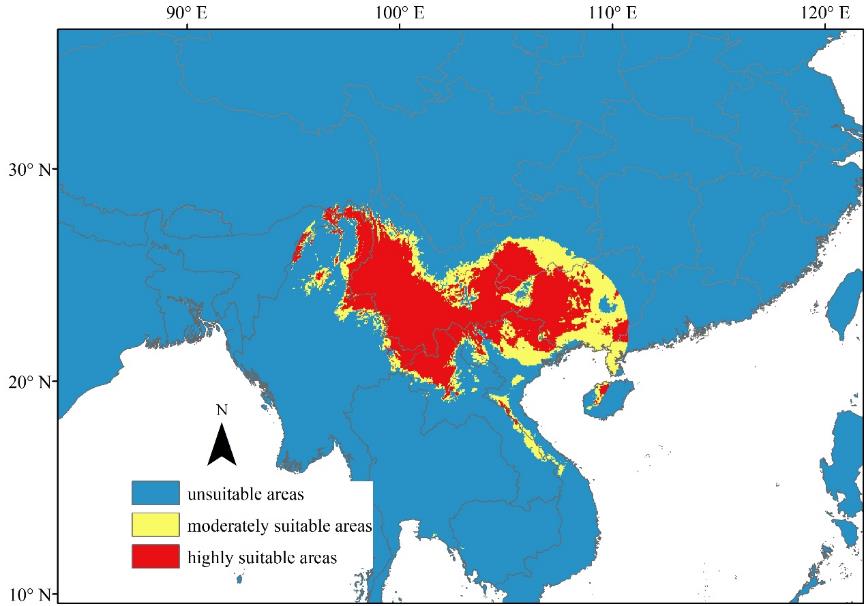


**Figure S53.** The distribution of *Notobitus excellens*

29. *Notobitus meleagris*

For *Notobitus meleagris*, selected environmental factors were BIO02, BIO03, BIO05, BIO07, BIO08, BIO11, BIO12, BIO13, BIO15, BIO17, BIO18, BIO19. ENMeval results indicated that the best parameter combination is LQHP+3 (Figure S53). Under these parameters, the AUC of MaxEnt model is 0.89. Species distribution areas showed on Figure S54.


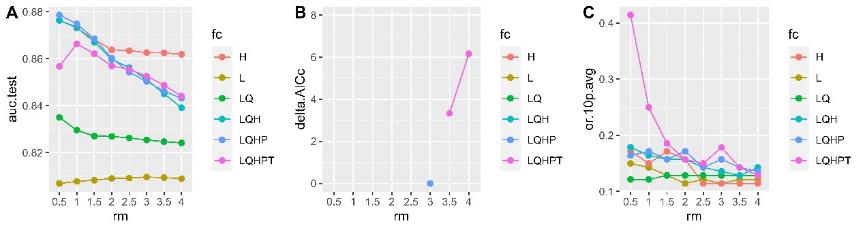


**Figure S54.** ENMeval results for *Notobitus meleagris*


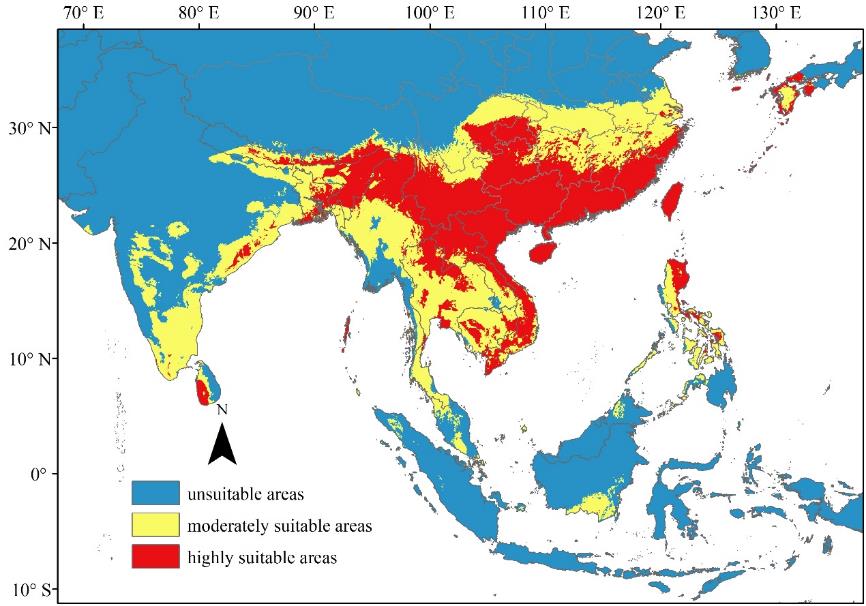


**Figure S55.** The distribution of *Notobitus meleagris*

30. *Notobitus montanus*

For *Notobitus montanus*, selected environmental factors were BIO02, BIO03, BIO04, BIO05, BIO06, BIO08, BIO12, BIO15, BIO18. ENMeval results indicated that the best parameter combination is L+1 (Figure S55). Under these parameters, the AUC of MaxEnt model is 0.71. The model failed to simulated the species distribution, its distribution will simulated by point to grids method.
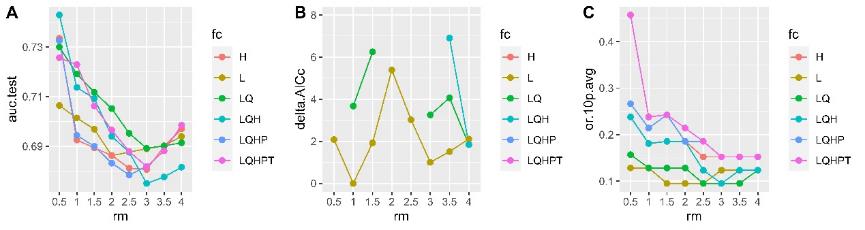


**Figure S56.** ENMeval results for *Notobitus montanus*

31. *Notobitus sexguttatus*

For *Notobitus sexguttatus*, selected environmental factors were BIO02, BIO03, BIO05, BIO08, BIO11, BIO12, BIO17, BIO18. ENMeval results indicated that the best parameter combination is LQHPT+4 (Figure S56). Under these parameters, the AUC of MaxEnt model is 0.81. Species distribution areas showed on Figure S57.


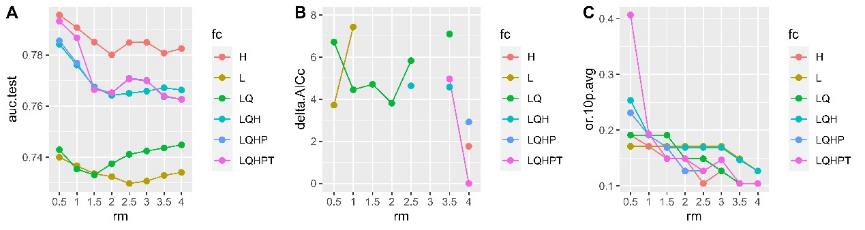


**Figure S57.** ENMeval results for *Notobitus sexguttatus*


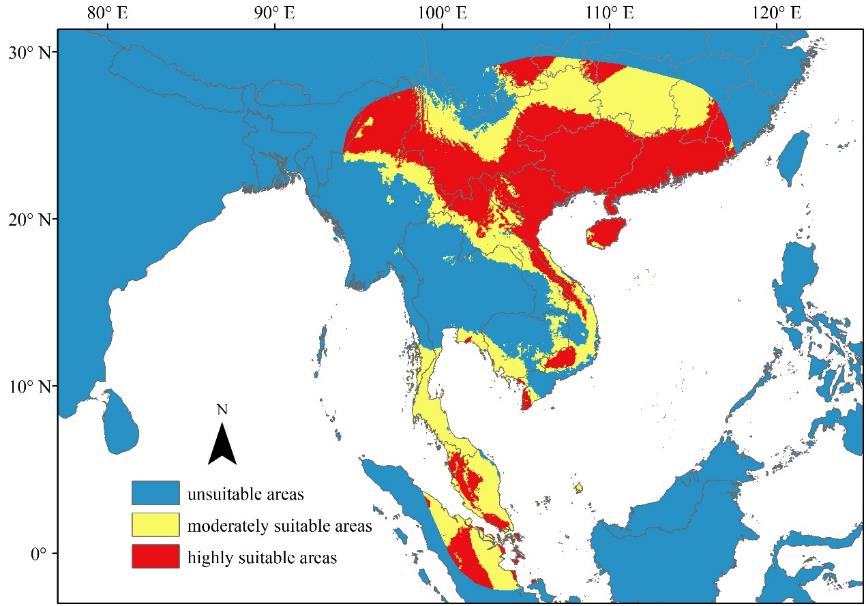


**Figure S58.** The distribution of *Notobitus sexguttatus*

32. *Paramarcius puncticeps*

For *Paramarcius puncticeps*, selected environmental factors were BIO02, BIO03, BIO04, BIO08, BIO10, BIO12, BIO15. ENMeval results indicated that the best parameter combination is LQ+0.5 (Figure S58). Under these parameters, the AUC of MaxEnt model is 0.88. Species distribution areas showed on Figure S59.


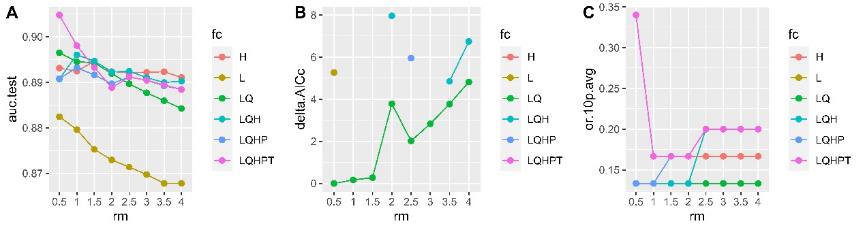


**Figure S59.** ENMeval results for *Paramarcius puncticeps*


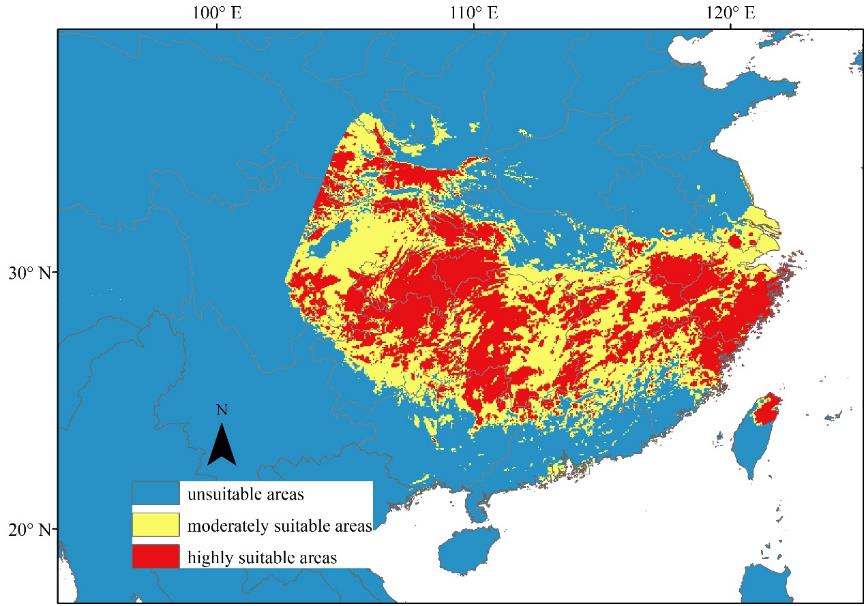


**Figure S60.** The distribution of *Paramarcius puncticeps*

33. *Paterculus elatus*

For *Paterculus elatus*, selected environmental factors were BIO02, BIO03, BIO07, BIO08, BIO09, BIO10, BIO12, BIO16, BIO17, BIO18, BIO19. ENMeval results indicated that the best parameter combination is LQ+3.5 (Figure S60). Under these parameters, the AUC of MaxEnt model is 0.91. Species distribution areas showed on Figure S61.


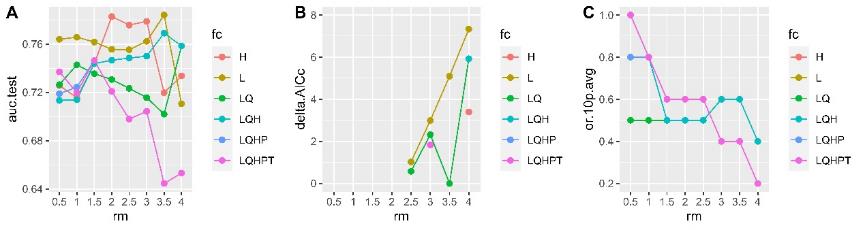


**Figure S61.** ENMeval results for *Paterculus elatus*


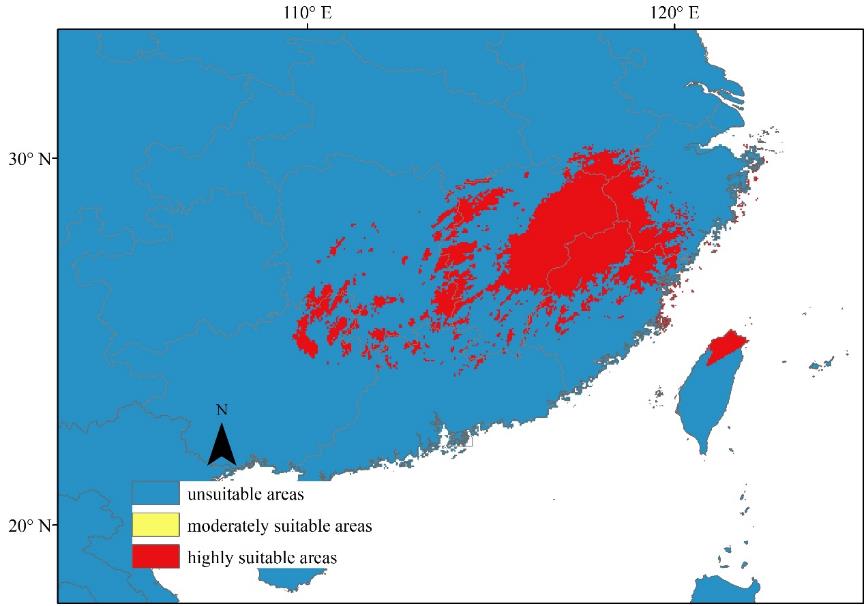


**Figure S62.** The distribution of *Paterculus elatus*

34. *Phenacantha bicolor*

For *Phenacantha bicolor*, selected environmental factors were BIO02, BIO04, BIO06, BIO10, BIO12, BIO13, BIO15, BIO18. ENMeval results indicated that the best parameter combination is LQ+1.5 (Figure S62). Under these parameters, the AUC of MaxEnt model is 0.93. Species distribution areas showed on Figure S63.


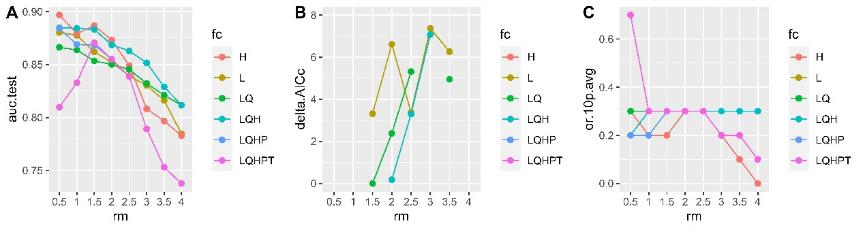


**Figure S63.** ENMeval results for *Phenacantha bicolor*


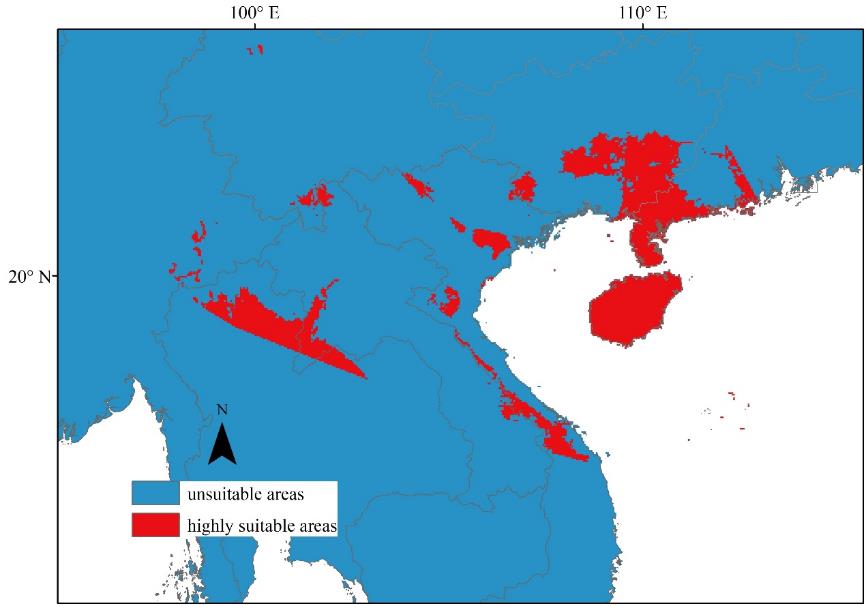


**Figure S64.** The distribution of *Phenacantha bicolor*

35. *Pirkimerus japonicus*

For *Pirkimerus japonicus*, selected environmental factors were BIO02, BIO03, BIO04, BIO05, BIO06, BIO08, BIO13, BIO15, BIO19. ENMeval results indicated that the best parameter combination is LQHPT+3 (Figure S64). Under these parameters, the AUC of MaxEnt model is 0.76. The model failed to simulated the species distribution, its distribution will simulated by point to grids method.


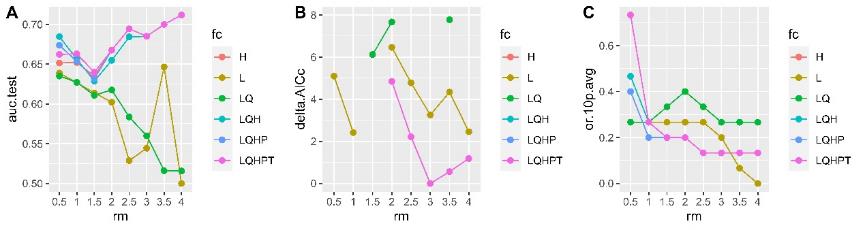


**Figure S65.** ENMeval results for *Pirkimerus japonicus*

36. *Tuberculiformia subinermis*

For *Tuberculiformia subinermis*, selected environmental factors were BIO01, BIO02, BIO06, BIO07, BIO09, BIO10, BIO14, BIO15, BIO18. ENMeval results indicated that the best parameter combination is LQ+1 (Figure S65). Under these parameters, the AUC of MaxEnt model is 0.91. Species distribution areas showed on Figure S66.


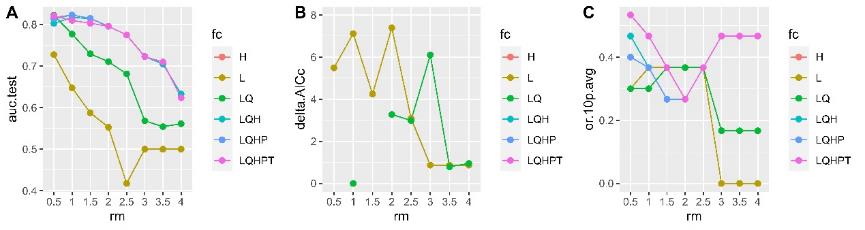


**Figure S66.** ENMeval results for *Tuberculiformia subinermis*


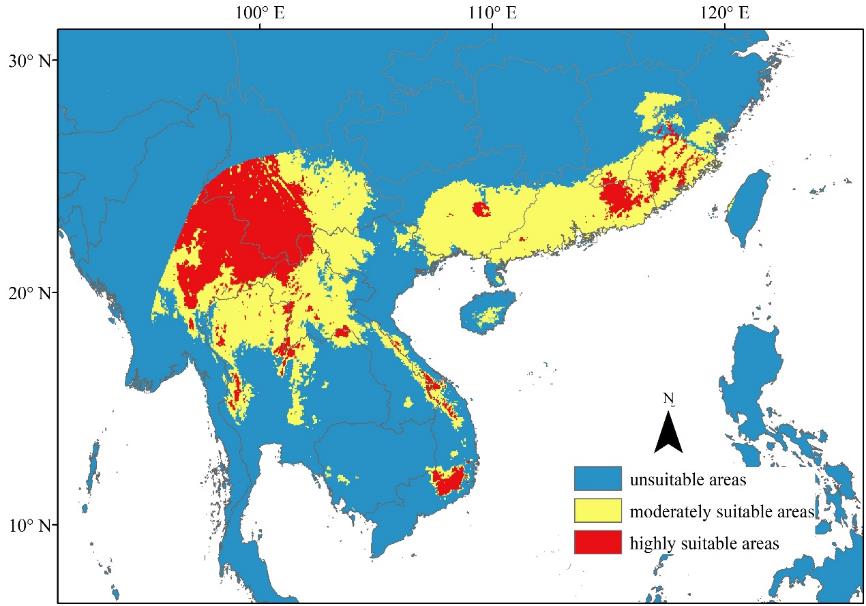


**Figure S67.** The distribution of *Tuberculiformia subinermis*

37. *Vitruvius insignis*

For *Vitruvius insignis*, selected environmental factors were BIO01, BIO08, BIO09, BIO18, BIO19. ENMeval results indicated that the best parameter combination is L+1 (Figure S67). Under these parameters, the AUC of MaxEnt model is 0.75. The model failed to simulated the species distribution, its distribution will simulated by point to grids method.


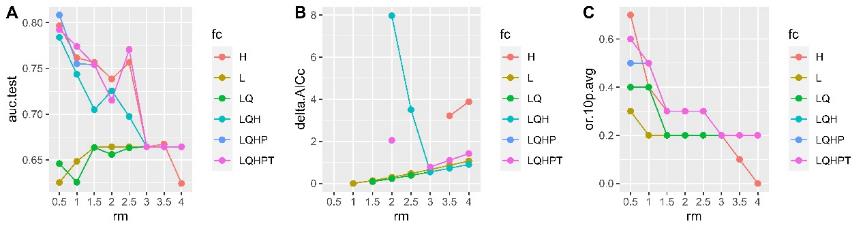
**Figure S68.** ENMeval results for *Vitruvius insignis*

38. *Yemmalysus parallelus*

For *Yemmalysus parallelus*, selected environmental factors were BIO02, BIO05, BIO07, BIO08, BIO11, BIO12, BIO15, BIO17, BIO18. ENMeval results indicated that the best parameter combination is LQHPT+3.5 (Figure S68). Under these parameters, the AUC of MaxEnt model is 0.87. Species distribution areas showed on Figure S69.


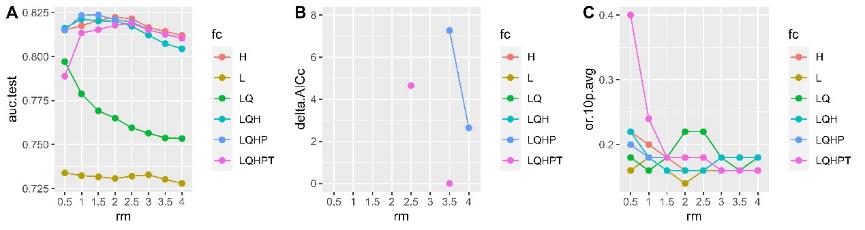


**Figure S69.** ENMeval results for Yemmalysus parallelus


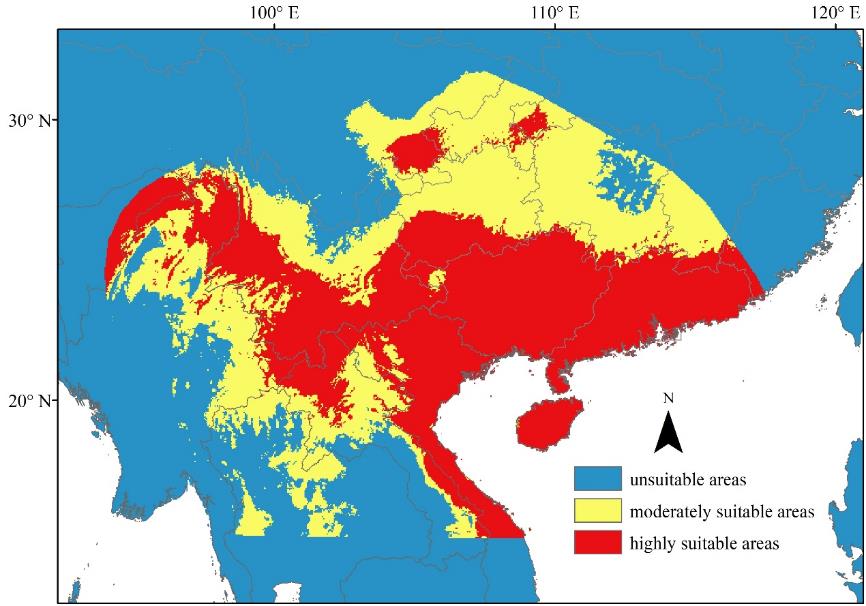


**Figure S70.** The distribution of *Yemmalysus parallelus*

**Reference**

Chen, Z.-y. (1986). A new species of *Notobitus* from Guangdong Province(Hemiptera: Coreidae). Acta Ecologica Sinica, 11(3), 325-326.

Chen, Z.-y. (1989). The Hemiptera on bamboos from Guangdong Province. Journal of Bamboo Research, 008(003), 58-60.

Fan, Z.-h., & Liu, G.-q. (2009). The Genus *Aenaria* Stal, 1876 in China (Hemiptera, Pentatomidae). Acta Zootaxonomica Sinica.

Hsiao, T.-Y. (1977). A handbook for the determination of the Chinese Hemiptera-Heteroptera: Nankai Univ.

Jiang, K., Chen, J., & Bu, W. (2022). A New Species in *Notobitiella* Hsiao in China Confirmed by Morphological and Molecular Data (Hemiptera: Heteroptera: Coreidae). Insects, 13(5), 411.

Ren, S.-z. (1983). Notes on the genus Manocoreus Hisao from China (Heteroptera: Coreidae). Entomotaxonomia(04), 59-63.

Xu, T.-s., & Wang, H.-j. (2004). Main Pests of Bamboo in China: China Forestry Publishing House.

Zhang, S.-m. (1995). Economic insect fauna of China: Science Press.

Zheng, L.-Y. (1994). Heteropteran Insects (Hemiptera) Feeding on Bamboos in China. Annals of the Entomological Society of America, 87(1), 91-96.
